# A Novel Bioreactor System for the Assessment of Endothelialization on Deformable Surfaces

**DOI:** 10.1038/srep38861

**Published:** 2016-12-12

**Authors:** Björn J. Bachmann, Laura Bernardi, Christian Loosli, Julian Marschewski, Michela Perrini, Martin Ehrbar, Paolo Ermanni, Dimos Poulikakos, Aldo Ferrari, Edoardo Mazza

**Affiliations:** 1 ETH Zurich, Laboratory of Thermodynamics in Emerging Technologies, Sonneggstrasse 3, 8092 Zurich, Switzerland; 2 ETH Zurich, Institute for Mechanical Systems, Leonhardstrasse 21, 8092 Zurich, Switzerland; 3 ETH Zurich, Laboratory of Composite Materials and Adaptive Structures, Department of Mechanical and Process Engineering, Tannenstrasse 3, CH-8092 Zurich, Switzerland; 4 University Hospital Zurich, Department of Obstetrics, Zurich, Switzerland; 5 Empa, Swiss Federal Laboratories for Materials Science & Technology, Überlandstr. 129, 8600 Dübendorf, Switzerland

## Abstract

The generation of a living protective layer at the luminal surface of cardiovascular devices, composed of an autologous functional endothelium, represents the ideal solution to life-threatening, implant-related complications in cardiovascular patients. The initial evaluation of engineering strategies fostering endothelial cell adhesion and proliferation as well as the long-term tissue homeostasis requires *in vitro* testing in environmental model systems able to recapitulate the hemodynamic conditions experienced at the blood-to-device interface of implants as well as the substrate deformation. Here, we introduce the design and validation of a novel bioreactor system which enables the long-term conditioning of human endothelial cells interacting with artificial materials under dynamic combinations of flow-generated wall shear stress and wall deformation. The wall shear stress and wall deformation values obtained encompass both the physiological and supraphysiological range. They are determined through separate actuation systems which are controlled based on validated computational models. In addition, we demonstrate the good optical conductivity of the system permitting online monitoring of cell activities through live-cell imaging as well as standard biochemical post-processing. Altogether, the bioreactor system defines an unprecedented testing hub for potential strategies toward the endothelialization or re-endothelialization of target substrates.

Statistical predictions for the ageing population of Western Countries foresee a dramatic increase of cardiovascular patients in the next two decades, which will manifest itself as a rapidly growing public health issue with significant economic impact^1^. In particular, almost 40 million people are expected to suffer of heart failure and related complications^2^.

Heart transplantation is the current treatment option in case of severe heart failure, however it is limited by donor heart availability and patient eligibility^3^. Recent developments in circulatory support system technology have established ventricular assist devices (VADs) as a viable bridge-to-transplant solution^4^. The further development of VADs into destination therapy, and thus their deployment as a substitute for transplantation, is hindered by the excessive incidence of device-related adverse events^5^. One of the main complications in state-of-the-art VADs is blood coagulation triggered by the contact between blood and artificial materials comprising the device which is partially restrained by intense administration of blood thinners in turn exposing the patient to hemorrhagic events^6^,^7^,^8^.

The long term integration of cardiovascular devices can be obtained through the formation of a living protective layer, generated by autologous endothelial cells (ECs), at the implant’s luminal surface^9^. Several strategies have been proposed to address the process of endothelialization of artificial materials (i.e. metal alloys, plastic polymers, and elastomers). These include the chemical modification of synthetic interfaces in contact with blood^10^, the surface structuring with rationally engineered topography^11^,^12^,^13^, or the biological functionalization with intervening layers of basal matrix components or biological molecules promoting the binding and proliferation of ECs^14^. The common goal of these approaches is to promote specific endothelial activities, overall supporting the generation and long-term maintenance of a functional monolayer, in order to support the establishment of local homeostasis and prevent the direct contact between blood and artificial materials^15^,^16^. Despite significant technological advancements, a viable endothelialization protocol is still missing. The luminal endothelialization of cardiovascular implants remains anecdotal and largely insufficient to cope with the high number of post-deployment complications in cardiovascular patients^17^,^18^.

Endothelialization strategies are initially developed based on *in vitro* tests, which often fail to recapitulate the complex environment experienced by ECs at the interface between blood and synthetic materials *in vivo*^9^. Perhaps the most important regulator of endothelial function, from adhesion to polarization, stems from the hemodynamic conditions generated by the local pattern of blood flow, the wall geometry, and the deformability of the wall materials^19^. The temporal variations and absolute magnitude of flow-generated wall shear stress (WSS) and wall deformation (WD) showed a critical impact on all tested ECs activities^20^,^21^,^22^. In particular, the migration and polarization of ECs are directly modulated by the direction and time pattern of flow^23^,^24^. The stability of substrate adhesions and Vascular Endothelial Cadherin (VEC)-based cell-to-cell junctions is controlled by the absolute WSS value^25^,^26^. The EC migration potential upon wound healing is regulated by the flow direction and the resulting WSS values^11^,^23^. The monolayer response to inflammatory insults similarly depend on flow directionality and WSS. EC polarization is dictated by the direction of flow and of substrate deformation^27^.

Partial access to physiological hemodynamic conditions has been introduced through bioreactors able to produce dynamic patterns of WSS^23^,^28^, uni- or biaxial stretch (summarized recently in refs 29 and 30) or some combination of these^31^,^32^. Only few examples reported the concomitant application of flow and uniaxial strain^33^,^34^,^35^,^36^ on ECs but did not explore WSS values higher than 2 Pa. Existing devices do not allow to study the endothelial response to WD and WSS in a range comparable to that experienced by ECs at the luminal surface of passive arterial grafts or active deformable elements of VADs^30^. Specifically, WSS in the range up to 10–15 Pa are present in VADs^37^,^38^ in regions identified as possible sources of thrombus formation^37^. Pulsatile VADs generate complex pattern of WSS and WD on the propulsion membrane with values close to the physiological range, i.e. up to 15% strain for WD (see e.g. ref. 39) and 6 Pa for WSS^40^. The two stimuli can be reproduced in existing bioreactors but with limitations in the magnitude: Amaya *et al*.^41^ developed a combined system for which WD is up to 20% but WSS is limited in the range between 0 and 2 Pa; the device by Dancu and Tarbell^42^ is also limited in WSS (max 2 Pa). The company Flexcell proposes a system where the maximum strain applicable is 4% (http://www.flexcellint.com/FlexFlow.htm).

A custom-developed, parallel plate flow bioreactor yielding extended control over physiological and supraphysiological WSS values (up to 12 Pa) was recently introduced^43^. This bioreactor enabled the study and validation of endothelialization strategies under WSS conditions reproducing those expected in pumping systems such as VADs^44^. Endothelial response to WD in the range of those experienced at the luminal surface of passive arterial grafts or active deformable elements of VADs as well as the effect of complex time patterns of combined WSS and WD were largely neglected due to the challenges connected to the development of a reliable bioreactor with such capabilities^30^.

We hereby introduce a novel, custom-designed flow bioreactor system, enabling the long-term *in vitro* testing of endothelialization strategies for a broad range of complex realistic physiological and supraphysiological flow conditions. The system enables the independent control of WSS (up to 20 Pa) and WD (with uniaxial and biaxial strain up to 20%) yielding a wide range of spatiotemporal gradients of mechanical stimulation on endothelial monolayers, which encompass the hemodynamic conditions experienced at the luminal interface of VADs. The system is optically conductive and therefore accessible by high-resolution microscopes for online inspection of endothelial activities. The overall design and implementation of the system presented and its validation is exemplified with respect to the assessment of endothelialization of artificial materials obtained using primary human endothelial cells (HUVECs) which are exposed to a range of stimulations for prolonged periods of time (up to 24 h). These new experiments also reveal novel insights into the response of ECs to overlapping gradients of WSS and WD requiring further dedicated investigations.

## Results

### Working principle of the reactor system

The system applies a cyclic predefined state of deformation to an elastomeric membrane covered by endothelial cells (ECs). This Wall Deformation (WD) displaces the membrane generating a partial obstruction of the flow in the chamber. In this manner the ECs are exposed to a controlled time-variable flow field leading to specific pattern of Wall Shear Stress (WSS) on the cell layer. The realized concomitant and time-variable WD and WSS, are representative of a variety of conditions experienced by ECs in heart ventricles, large vessels, and cardiovascular devices.

### Design and operation

**Figure 1:**
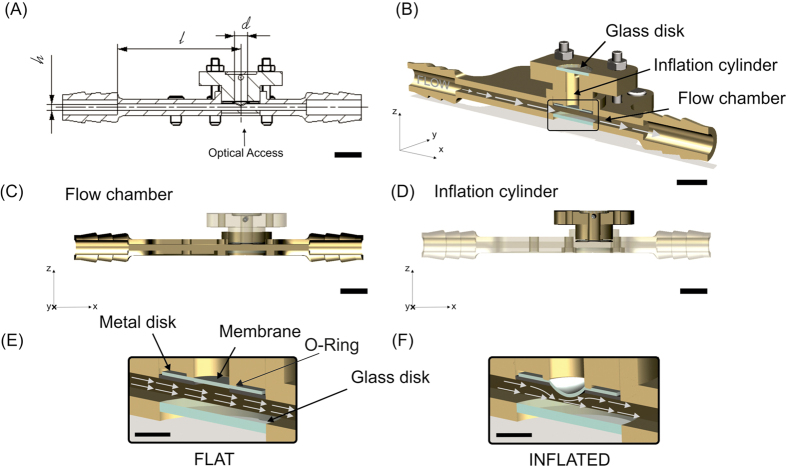
Design of the System. (**A**) Bioreactor chamber dimensions. l = 47 mm (entrance length); h = 2.5 mm (chamber height); w = 6 mm (chamber width, not shown) and d = 5 mm (diameter of the inflated membrane). (**B**) Global cross section view of the bioreactor. (**C**) View of the reactor with transparent inflation part. (**D**) View of the reactor with transparent flow part. The insets show the membrane (clamped in between Metal disk and O-Ring) in its flat state (**E**), corresponding to the minimum shear stress, and its maximum inflated state (**F**) that corresponds to the maximum shear stress conditions. The scale bars in panels (**A**–**D**) correspond to 10 mm and the scale bars in panels (**E**) and (**F**) to 5 mm, respectively.

The reactor was designed to generate a range of complex combinations of mechanical loading through the independent control of WSS and cyclic mechanical stretch (i.e. WD) on ECs (Fig. 1). The dynamic ranges of WSS and WD were selected to encompass the physiological values experienced by ECs in the human circulation (i.e. WSS values up to 6 Pa and WD values up to 10%). In addition, the bioreactor was designed with the unique capability of generating supraphysiological hemodynamic conditions similar to the ones expected at the luminal surface of VADs (i.e. WSS higher than 6 Pa and WD up to 20%). Finally, the materials and the overall bioreactor geometry were chosen to maximize optical access to the region housing the ECs (Fig. 1).

Figure 1 schematically illustrates the overall reactor design. Two main, independently-controlled compartments are displayed (Fig. 1C,D). The first corresponds to a flow chamber housing the ECs during the flow- conditioning experiments (Fig. 1C). The chamber features external dimensions of 25 × 60 mm and an internal rectangular cross section of 6 × 2.5 mm^2^. The inlet and outlet of the flow chamber are placed at the extremities and connect to the peristaltic pumping device to generate a fully-developed flow of cell culture medium on the central region of the chamber and therefore yielding a desired WSS on EC monolayers.

The second element is an inflation system that actuates cyclic stretch on the deformable membrane covered by ECs (Fig. 1D). The membrane is comprised of a PDMS-based elastomer, which faces the flow chamber and supports the endothelial monolayer at its luminal surface. The membrane inflation system is composed of a cylinder of 15 mm outer diameter and 5 mm inner diameter fixed to the flow chamber by 4 screws (Fig. 1B). The volume of liquid (i.e. PBS) inside the cylinder is controlled with a syringe pump receiving online feedback from a pressure sensor. The luminal end of the cylinder extends towards the flow section and is separated from it by the interfacing deformable membrane. Therefore, the hydrostatic pressure in the cylinder actuates the cyclic inflation of the membrane during the experiment. In this manner, a controlled state of biaxial deformation is applied to the ECs (Fig. 1E,F).

### Control and Validation

**Figure 2:**
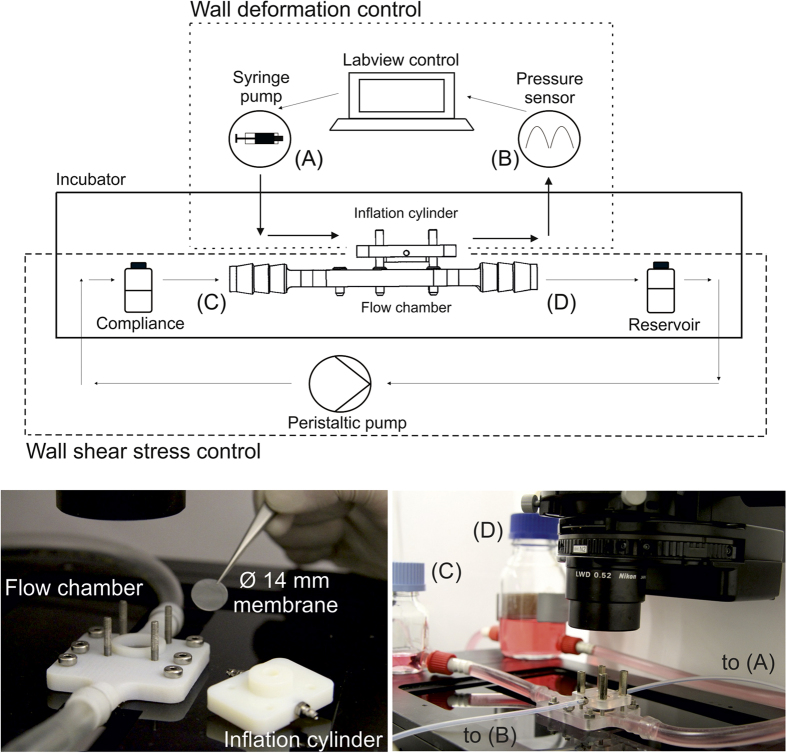
Control of actuation. The set-up consists of two independently controlled components. The inflation cylinder is actuated by a syringe pump (**A**). The pressure in the inflation cylinder is monitored by a sensor (**B**) and controlled via a custom-developed LabView software. The flow chamber is actuated by a peristaltic pump connected to a compliance (**C**) and a reservoir (**D**).

**Figure 3:**
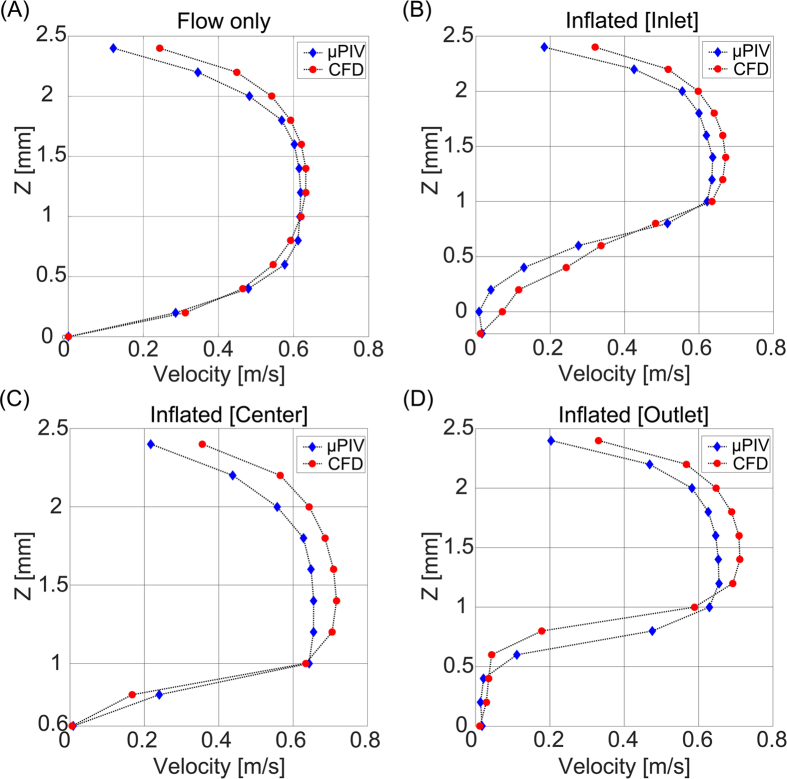
Validation of the velocity field. Comparison of Computational Fluid Dynamics (CFD) and Microparticle Image Velocimetry (μPIV) flow profiles at 0.4 l/min flow. (**A**) CFD (red circles) and μPIV (blue rhombus) profiles for the flat membrane configuration. (**B–D**) Corresponding profiles for the configuration with membrane inflated to 20% extension (i.e. 720 mbar) at the inlet (**B**), center (**C**), and outlet (**D**), respectively.

**Figure 4:**
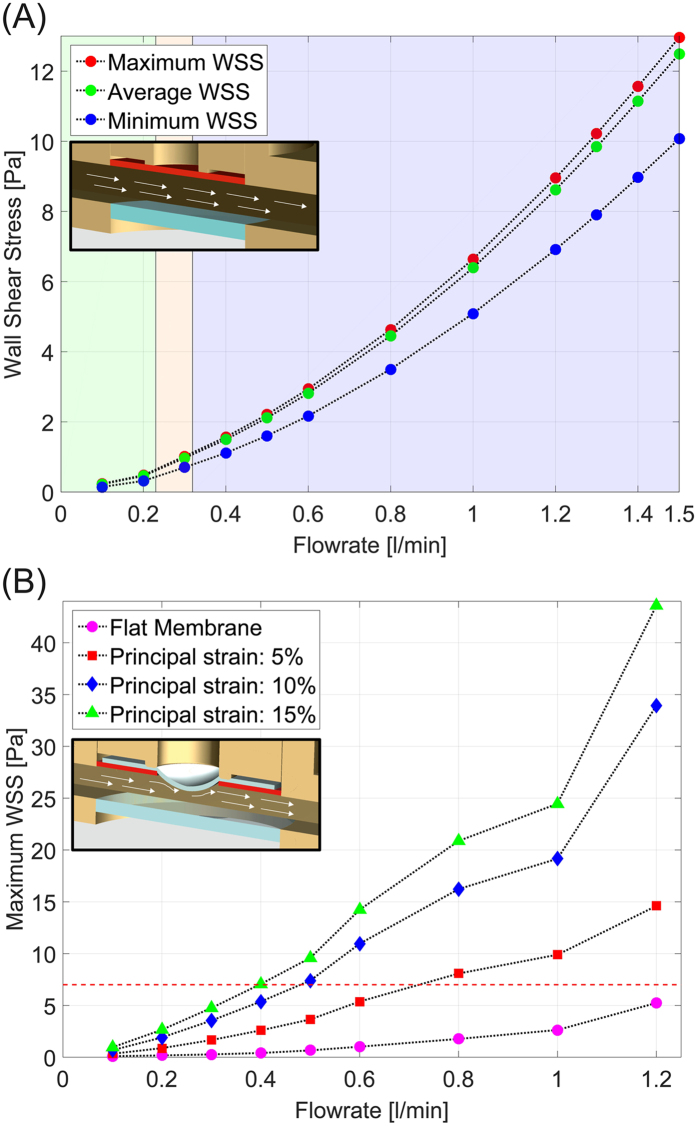
WSS profiles in the reactor. (**A**) Maximum (red circles), average (green circles), and minimum (blue circles) WSS at the surface of the deformable membrane as a function of pure flow rate (flow rates from 0.1 l/min to 1.5 l/min) in the flat membrane configuration. (**B**) Maximum WSS at the surface of the deformable membrane as function of the flow rate and for different membrane strain conditions ranging from flat (pink circles) up to surface strain of 15% (green triangles).

The reactor system is actuated by two independently-controlled pumps that can operate individually (Fig. 2). When running on a single pump the reactor performs either as flow chamber exposing the endothelial monolayer to defined WSS, or alternatively as pure stretching device yielding uniaxial and biaxial WD. The validation of the device reported in the following was performed first for the single components operating individually and, in a second phase, for the combined modality of operation. In the flow-only configuration, omitting the metal disk allows to generate higher WSS levels. The components and the assembly of the reactor are illustrated in Supplementary Video 1. The dynamics in the flow chamber were characterized by a computational fluid dynamics simulation (CFD, see Methods and Supplementary Video 2), which was experimentally supported by corresponding microparticle image velocimetry (μPIV) measurements in the channel (Fig. 3). For the configuration with no metal disk and a flat membrane, we simulated steady-state flow patterns within the fluidic channel under the assumption of a fully-developed flow. The good agreement with the results from the μPIV measurement (Fig. 3A) shows that the system is capable of applying up to 13 Pa of WSS on the cells located in the central housing of the channel in this configuration (Fig. 4A).

These results demonstrate that the flow in the reactor is suitable for testing the effect of both physiological and supraphysiological flow conditions on ECs. In addition, to exclude possible fluid flow fluctuations generated by the peristaltic pump, a turbine flow sensor was inserted in the flow channel. The experimental measurements retrieved confirmed that flow fluctuations did not exceed 15% of the set value during long-term operation of the flow system (Supplementary Figure 1).

For the various membrane configurations with the metal disk present, different conditions apply. During the stretching loop, the deformable PDMS membrane cycles between two states: the flat state (Fig. 1E) during which the substrate is in its reference configuration (i.e. no WD), and the inflated state (Fig. 1F) during which the maximal imposed stretch is reached (i.e. maximal WD). To regulate the time history of fluid pressure applied in the inflation system over the whole duration of a conditioning experiment a dedicated control algorithm was developed. The stretch of the membrane is actuated by the movement of liquid in and out of the inflation cylinder establishing a pressure load on the elastomer. In particular, the flat and the inflated states of the membrane correspond to the start and end positions of the syringe pump piston, respectively (Supplementary Figures 2 and 3). The level of deformation of the membrane depends on the pressure generated in the inflation cylinder and is controlled based on corresponding model equations. The cyclic inflation (from flat to inflated) occurs at a defined frequency in the range between 0.85 and 1.1 Hz. The maximum inflation as well as the inflation frequency are selected for each experiment through the corresponding parameters in the control software (Supplementary Figures 3 and 4).

Supplementary Figure 2 summarizes the results of a validation test for the inflation system and the corresponding control algorithm. For this test the system was set to reach 220 mbar yielding a maximal principal strain of (approximately) 8% with a cyclic stretch frequency of 1 Hz. These conditions were selected to represent elevated physiological values of stretch and frequency. During the test the pressure was measured by a membrane-deformation pressure sensor with a sampling frequency of 10 Hz that was connected to the inflation chamber and supplied to the control algorithm. In the control feedback loop the software compared the maximum pressure acquired in a time frame of 10 s to the target maximum pressure and used the magnitude of the difference (with a 5 mbar tolerance) to adjust the end position of the syringe pump piston (Supplementary Figure 4). The effect of the adjustment was then measured (with an accuracy of ±1.5%) over the next 10 s, before starting a new control loop. At the same time the starting position of the syringe pump piston defines the reference configuration in which the deformable membrane should be in a flat state. In case that at this point the measured reference pressure was negative (<0 mbar), the starting position of the piston was adjusted. In all, these results demonstrate that the system was able to constantly maintain the pressure between the set values (i.e. 0 ± 5 and 220 ± 5 mbar) for more than 10^5^ cycles thus establishing the long-term stability of the cyclic stretch actuation of the reactor.

**Figure 5:**
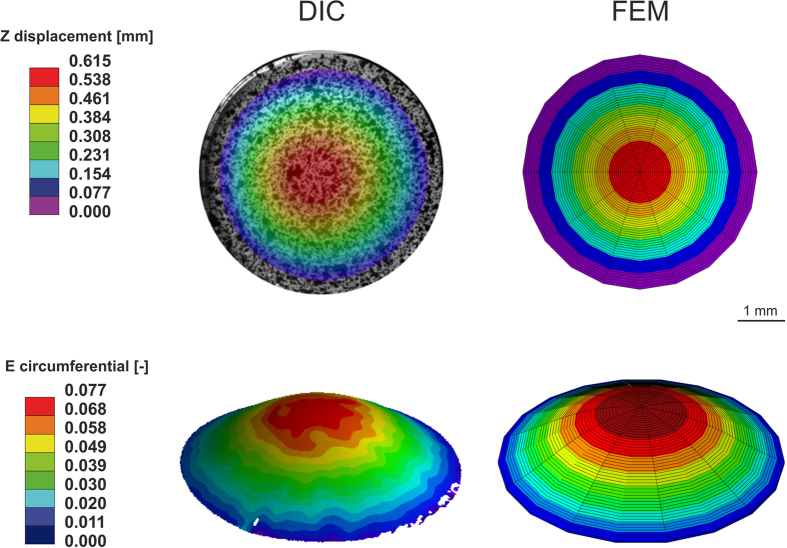
Validation of the inflation cylinder. Comparison between Digital Image Correlation (DIC) results for a 500 μm thick membrane loaded with 220 mbar pressure and the corresponding Finite Element Model (FEM). Top: Z displacement. The max displacement given by the DIC measurement is 0.615 mm; the predicted FE displacement is 0.614 mm. Bottom: Strain. The apex strain given by the DIC measurement is 7.7%; the predicted FE maximum strain is 8.4%.

Actuation of the inflation device yielding multiaxial stretch of the deformable membrane was validated through a finite element (FE) model yielding the strain field on the membrane for each combination of thickness and pressure, as well as the maximum membrane apex displacement. These results were directly compared to measurements obtained by digital imaging correlation (DIC, see Materials and Methods). As reported in Fig. 5, good agreement in terms of both displacement and strain was reached. In particular, for the case of a 500 μm thick membrane inflated with 220 mbar, a deviation of 1 μm (corresponding to less than 1%) in the out-of-plane displacement was observed. Regarding the strain field, the discrepancy between the two reported methods was slightly larger (up to 1% strain) but still within the experimental uncertainties. These results demonstrate that the stretch component of the reactor is suitable for applying predefined deformation pattern and thus testing the effect of a wide range of WD (i.e. physiological and supraphysiological) on ECs.

When both components of the reactor are actuated, inflation of the deformable membrane into the flow chamber generates a dynamic combination of asymmetric WSS and multiaxial WD on the luminal surface of the deformable membrane. Specifically, the system cycles between the flat state (i.e. no WD, Fig. 1E) in which the surface of the deformable membrane only experiences the WSS imposed by the actuation of the flow channel, and a fully inflated state (i.e. maximal WD, Fig. 1F) in which the maximal WD is superimposed. The membrane bulging into the fluid stream interacts with the flow pattern to generate areas of high WSS on the upstream half of the deformable membrane and regions of recirculation and low WSS in the downstream half. The resulting redistribution of the WSS pattern upon the membrane inflation cycle was evaluated through *in silico* numerical simulations for various flow conditions and experimentally confirmed by μPIV measurements (Fig. 3). Importantly, the combined actuation of the two reactor systems creates areas of the deformable membrane which, in the fully inflated state, are exposed to higher (in the front) or lower (in the rear) values of WSS, as compared to those defined by the sole actuation of the flow channel with identical flow.

### Assessment of endothelialization

We next set to validate the reactor for the assessment of endothelialization under loading conditions simulating the values which are expected in regions of implanted VADs. In particular, during the cell-conditioning experiments, a time-dependent WSS and WD field were created on a fully differentiated and growth-arrested endothelium generated in static conditions at the luminal surface of the deformable membrane (Supplementary Figure 6). For this validation, flow values to yield maximum WSS encompassing the physiological and supraphysiological range (i.e. from 0.5 to 10) were selected. In addition, the inflation cylinder was actuated at a frequency of 1 Hz to reach a maximal pressure of 220 mbar, leading to an equibiaxial strain at the apex of approximately 8% in the fully inflated state. After starting the flow in the flow channel at the beginning of the conditioning experiment, the maximum pressure in the inflation cylinder was gradually increased to the target value, in order to avoid damage to the cells due to sudden stretch increase. The target pressure was typically reached within 5–10 min from the beginning of the experiment (Supplementary Figure 2).

At the end of the conditioning experiments, the quality and functionality of the endothelial monolayer covering the deformable membrane was evaluated. Specifically, values for cell coverage, density, and orientation were obtained by staining ECs for filamentous actin, Vascular Endothelial Cadherin (VEC) and cell nuclei^43^ (Supplementary Figure 6).

**Figure 6:**
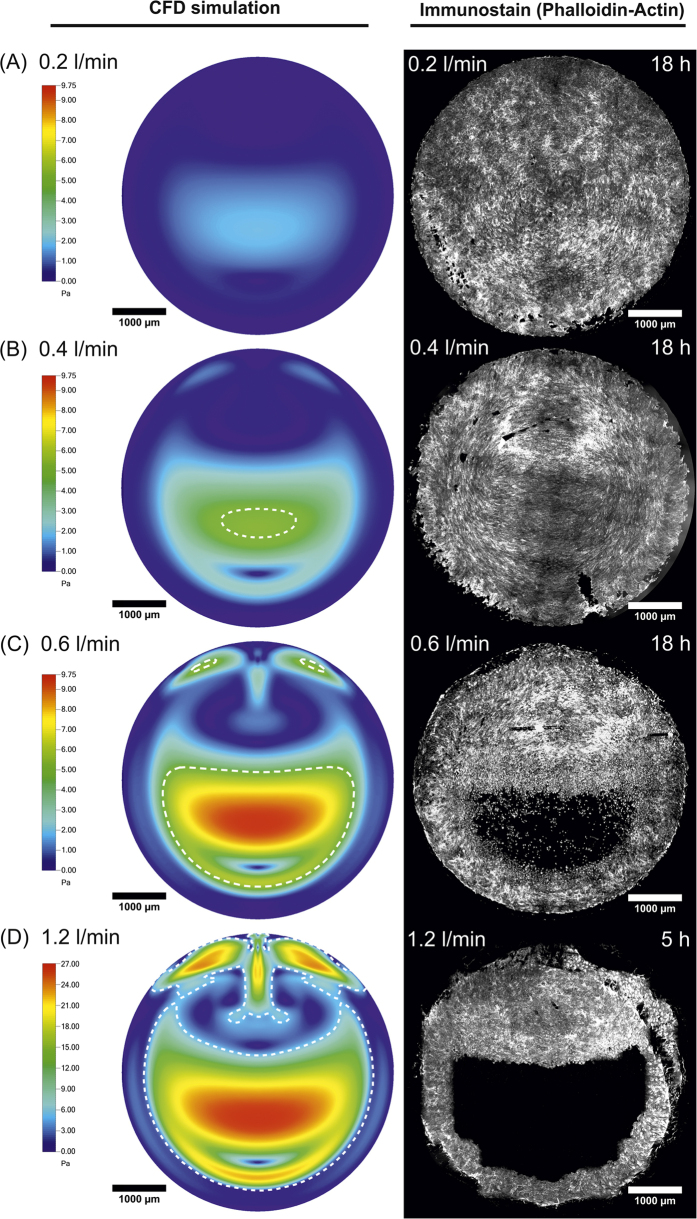
Endothelialization under complex hemodynamic conditions. (**A–D**) WSS patterns computed for the combined actuation of the flow channel and the inflation cylinder for an imposed flow of 0.2, 0.4, 0.6, and 1.2 l/min, respectively (left). The WSS maps correspond to the fully inflated state for a target of 8% cyclic biaxial strain. The dashed white line delimits regions that feature more than 5 Pa WSS. The right column reports the filamentous actin staining on HUVECs cells on a 5 mm diameter PDMS deformable membrane after 18 h conditioning. Note that at flow rates of 0.6 l/min (**C**) and higher (**D**) endothelialization was absent in the region of the membrane exposed to supraphysiological WSS. Corresponding high-resolution images are provided as Supplementary Figures 8–11.

**Figure 7:**
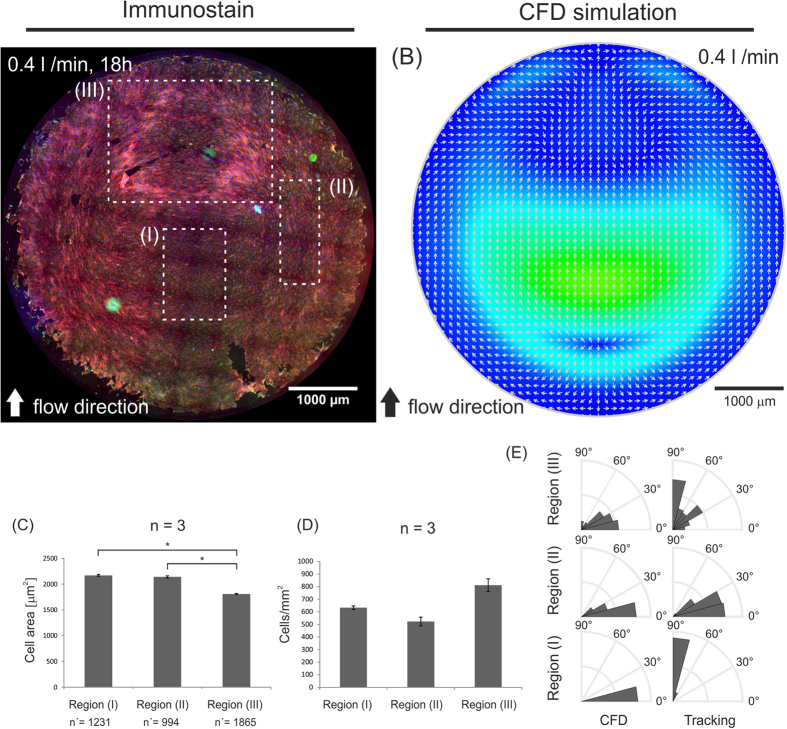
Effect of complex WSS and WD patterns on surface endothelialization. (**A**) Fluorescent images of an endothelium covering the deformable membrane after 18 h of flow conditioning at 0.4 l/min and membrane deformation at 8% maximal strain and 1 Hz frequency. (Red = Actin, Blue = Nuclei, Green = VEC). (**B**) Three regions of interest (I, II, and III) were selected corresponding to areas exposed to different WSS in the fully inflated state of the deformable membrane (Fig. 1F). (**C**) Corresponding measure for cell area and (**D**) cell density. Significant differences for the cell area are reported (*for α < 0.05). n denotes the number of independent experiments and n′ denotes the number of measured cells. Error bars correspond to the standard error of the mean. (**E**) The rose plots report the measured cell alignment (tracking) to the direction of flow as predicted by CFD analysis (CFD).

Endothelial monolayers exposed to WD under flow rates yielding physiological WSS values along the entire inflation cycle of the deformable membrane (i.e. 0.2 and 0.4 l/min) were able to withstand the load and maintain full coverage and connectivity over the entire target substrate (Fig. 6A,B). Importantly, higher flow rates (i.e. 0.6 and 1.2 l/min, Fig. 6C,D) generated areas of the deformable membrane, which were exposed to supraphysiological WSS values (between 6 and 26 Pa) values in the fully inflated state (Fig. 4). In these regions endothelialization was compromised, creating areas of the deformable membrane partially or fully depleted of cells (Fig. 6C,D). This process could be visualized through live-cell microscopy (Supplementary Videos 3 and 4). Time-lapses obtained at regions of supraphysiological WSS revealed that cell loss in these areas was the result of cell migration along a WSS gradients, with ECs moving toward regions of lower WSS (Supplementary Video 4). In all, this data confirms the viability of the system to study the effect of combined WSS and WD loading on the endothelialization of artificial materials showing a striking agreement between the computed values of WSS and the resulting effect on endothelialization (Figs 6 and 7). Furthermore, it suggests that the effect of the selected moderate values of circumferential stretch was not causing cell depletion if not combined with complex patterns of elevated WSS on the target surface. Emerging regions of supraphysiological WSS corresponded to areas of endothelialization loss and local gradients of WSS were able to direct cell migration.

**Figure 8:**
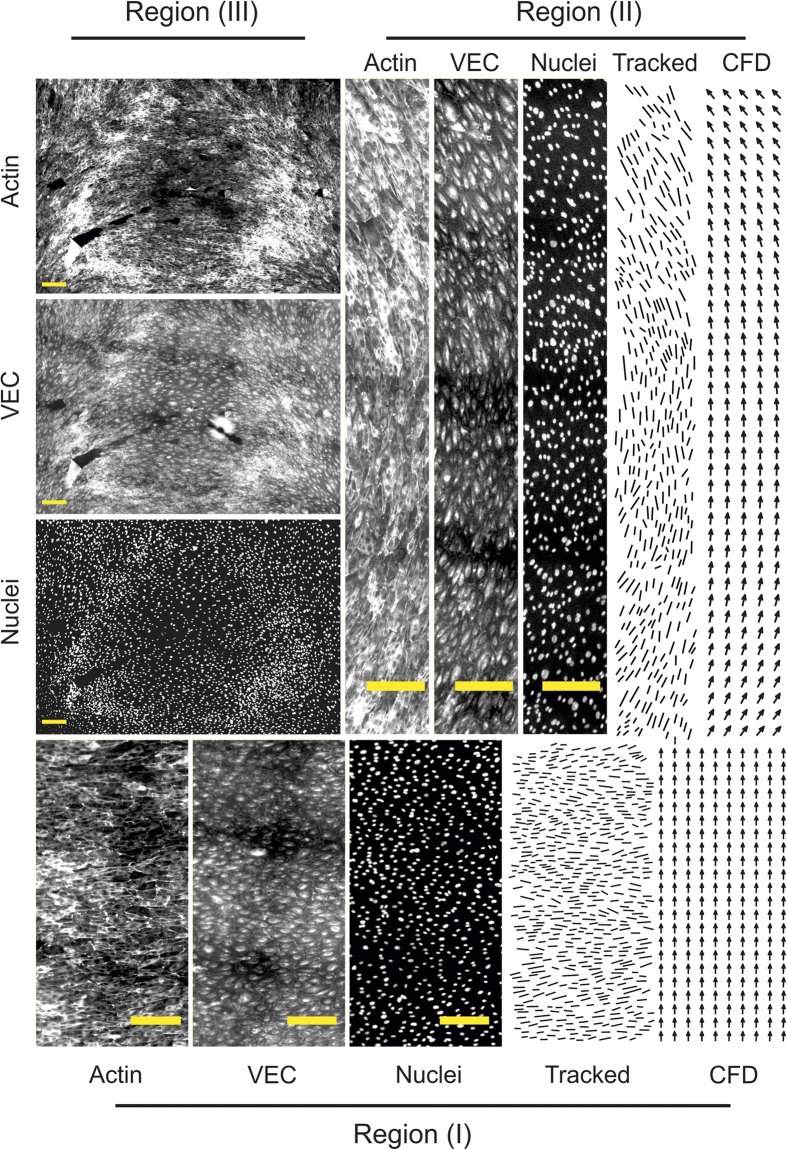
Endothelial cell alignment to flow. Zoomed immunofluorescent images of the endothelium in the regions of interest defined in Fig. 7 (Actin, VEC and Nuclei) and corresponding visualization of tracked cell alignment (line plot) and CFD flow direction predictions. Scale bar is 200 μm. Corresponding high-resolution images are provided as Supplementary Figures 12–20.

To obtain further insight into the effect of WSS gradients and of flow dynamics on the evolution of endothelial coverage of the target deformable target membrane we investigated the case of cells exposed to the combination of 0.4 l/min flow rate and 8% maximal deformation (Fig. 7). Here, the resulting patterns of cell alignment to flow could be distinguished in three regions which coincide with regions featuring different values of WSS (Figs 7 and 8). In regions of the monolayer which are exposed to low, physiological WSS values (i.e. between 0.1 and 4 Pa, Fig. 7A) all through the deformation cycle cells aligned along the direction of flow (flanking regions of the deformable membrane, Fig. 8). ECs exposed to high, physiological WSS (between 4 and 6 Pa) in the fully inflated state aligned perpendicular to the flow which is in agreement with what was previously reported (ref. 45 and Fig. 8). Here, cell density was increased, while cell area did not change significantly (Fig. 7C,D). Finally, the rear region of the deformable membrane, exposed to low WSS values and flow recirculation in the fully inflated state featured an area of the monolayer which was characterized by high density of cells forming a ring along the recirculation profile (Figs 7A and 8). These data indicate that a number of parameters have an active effect on the response of ECs to complex flow patterns, including absolute WSS and WD values, spatial and temporal gradients of WSS, and local flow direction.

## Discussion

In summary, the various elements of the work substantiate the introduced novel reactor system as a valuable platform to test the endothelialization of artificial materials under physiological and supraphysiological conditions of mechanical loading. In the reactor, primary human endothelial cells can be exposed to varying combinations of flow-generated WSS and WD for long periods of time and the maintenance of a confluent and functional endothelial monolayer can be assessed *in vitro*. The reactor’s optical access allows live cell and flow monitoring and thus the evaluation of endothelial cell response to flow and deformation.

The overall modular structure of the reactor enables independent control of the flow conditioning system (up to a target WSS of 20 Pa) and of the stretch device (up to a target deformation of 20%). Both elements can be fully operated in single modality, therefore yielding pure flow or pure stretch stimulation to endothelial cells (Figs 1 and 2). A specific novelty of the presented system is represented by its simultaneous actuation, which generates complex flow patterns on the deformable membrane supporting endothelial cells (Fig. 6). Owing to the geometry of the flow channel and of the deformable membrane inflation, the spatial variation of WSS during the inflation cycle can be reliably predicted by computational simulation, which are confirmed by the values obtained by experimental visualization (Figs 3, 4 and 6). Compared to other devices^41^,^42^, combined activation of flow and inflation systems allows to generate a wider range of conditions of cyclic WSS and multiaxial WD. Our system cannot provide homogeneous conditions of WSS and WD on the membrane of the bioreactor. The spatial variations of shear stress and strain are similar to those present in heart ventricles, in larger vessels as well as in VADs^37^,^38^,^39^,^40^. It thus allows investigating the influence of WD and WSS gradients on cell survival, functionality, shape, orientation and density.

We report experiments which confirm the viability of the system at hand to study the response of endothelia covering an artificial elastomeric membrane exposed to complex loading conditions for several hours (Fig. 6). The maintenance of the endothelial integrity and function is assessed both live to capture events such as cell migration or loss of adhesion and through end-point biological analysis to reveal the expression and localization of specific endothelial markers. Endothelial cells contacting flat artificial surfaces can withstand WSS values in the physiological range. In fact, the presence of physiological flow was found to enhance viability and has thus found application in tissue engineering^46^. In addition, at low to medium values (up to 5 Pa) the cells adapt to flow by orienting their shape along the direction of flow (Figs 7 and 8). At higher values endothelial cells initially orient perpendicular to flow, and subsequently lose adhesion and detach from the substrate leading to the formation of regions of the endothelium with compromised integrity and/or coverage^47^,^48^.

These characteristic endothelial responses to flow at the interface with artificial materials are well reproduced (Figs 6 and 7). The spatial and temporal variations of WSS generated by the superimposed inflation of the deformable membrane have a twofold effect. First, they create a time-dependent asymmetry of WSS on the surface of the deformable membrane. In the inflated state the upstream region and the cells adhering to it are exposed to higher levels of WSS than the downstream region where low WSS values and flow recirculation are generated (Fig. 6). The emergence of areas of supraphysiological WSS creates weak points of endothelialization where cells are lost and a direct contact between the flowing medium and the artificial materials is possible. Second, it provides an additional mechanical stimulation to cells adhering to the substrate, which have to adapt to the circumferential gradient of deformation. The overlapping of WSS and WD gradients may generate directional signals for migration, which can induce polarized cell movements yielding local changes in cell density (Fig. 7 and Supplementary Video 4).

Current endothelialization strategies that can be tested in the novel reactor system include rationally-designed surface topographies^43^,^45^ promoting endothelial adhesion and migration (which can be introduced on the elastomeric membrane by means of standard soft lithography^49^, coatings with matrix proteins cross-linked to the elastomer^50^, or intervening layers of polymers deposited by means of electrospinning and directly bonded to the PDMS^51^).

Altogether, the reactor is instrumental in decoupling the effects of a broad range of hemodynamic parameters on endothelial cells at the interface with artificial materials. Therefore, it provides an exciting platform for the study of fundamental cell activities and the validation of solutions aimed at creating a stable protective layer on artificial materials at the luminal surface of cardiovascular implants.

## Methods

### Fabrication and actuation

The combined flow-deformation bioreactor system can be divided in two main components, the flow chamber and the inflation cylinder (Figs 1 and 2). Both components were fabricated using a 3D-printing system (Objet 500 Connex™, Stratasys, PolyJet technology) employing the acrylic based polymer VeroWhitePlus RGD835 (Stratasys). Two round glasses (1 mm thickness) were glued to the inflation cylinder and to the flow chamber, respectively, in order to guarantee optical access to a deformable membrane placed at the interface between the two systems and supporting a layer of cells in endothelialization experiments (Fig. 1). The membrane, comprised of an elastomeric layer, was tightly pressed against a metal disk of outer and inner diameter of 15 mm and 5 mm diameter, respectively (Fig. 1). Sealing was obtained with an O-ring interposed between the membrane and the cylinder.

The flow of culture medium in the flow chamber was generated by a peristaltic pump (P1500, Harvard Apparatus) equipped with 7.9 mm inner diameter Tygon tubes (Harvard Apparatus). The pump was periodically calibrated and the flow rate for each experiment was selected using the pump control functions. The cell medium was stored in a reservoir upstream of the pump, while a compliance downstream of the pump, placed before the bioreactor, was used to dampen the intrinsic pulsatility of the peristaltic propulsion (Fig. 2). The liquid in the inflation cylinder (i.e. PBS) was actuated through a syringe pump (PSD/4 HAMILTON, Bonaduz) equipped with a 5 ml glass syringe. The pressure level of the PBS inside the inflation cylinder was measured through a pressure sensor (MPX 4250 AP, Freescale). The syringe pump was connected to a reservoir filled with PBS (Sigma).

### Fabrication of the deformable membrane

Polydimethylsiloxane (PDMS, Dow Corning, Sylgard 184) membranes with a thickness of 400 ± 20 μm and 500 ± 20 μm were fabricated by curing a 10:1 mixture of base to crosslinker in between two soap-dipped glass plates separated by a teflon spacer of the respective thickness. This method was adapted from ref. 52 and yielded membranes with an elastic modulus of 1.09 MPa^53^. The thickness h was verified by determining cross sections at 8 to 9 different, preselected points with a microscope in brightfield mode (LSM 5 Pascal, Carl Zeiss GmbH, Jena, Germany). The precision of the resulting measurement was approximately 0.01 mm.

### Optical transparency

The optical transparency across the reactor in the 450–780 nm optical window was assessed by means of a home-built inverted microscope equipped with a spectrometer (Princeton Instruments). The fully mounted bioreactor was placed with the luminal side of the deformable membrane facing the incoming light beam. A 10X air objective (Olympus MPlan N, 0.25 NA) was used to illuminate the sample with normal incidence angle and collect the reflected and scattered light. White light from a xenon lamp was brought to the set-up with an optical fiber (105 μm core size) in order to improve its spatial coherence. To calculate the absorption spectrum, the relation


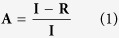


was used, where *A*, *I*, and *R* are the absorption, incident (excitation), and reflected spectra, respectively. Dark counts of the spectrometer and background spectra were subtracted from all the measured spectra.

### Cell culture

Human umbilical vein endothelial cells (HUVECs; ThermoFisher Scientific, USA) were grown in medium M200PRF supplemented with fetal bovine serum 2% v/v, hydrocortisone 1 mg/ml, human epidermal growth factor 10 ng/ml, basic fibroblast growth factor 3 ng/ml^54^,^55^, and heparin 10 mg/ml (all reagents from ThermoFisher Scientific) and were maintained at 37 °C and 5% CO_2_. All reported experiments were performed using cells with less than seven passages *in vitro*.

The deformable membranes were sterilized by overnight treatment with ethanol and rinsed three times with PBS before starting the coating procedure. The substrates were then coated with gelatin according to the protocol by ref. 56. The substrates were stored at 4 °C until the seeding of the cells. Cells were seeded on the substrates at high density (3.5–5 × 10^4^ cells/cm^2^). The substrates were then cultured for three days to generate a fully confluent and growth-arrested monolayer^57^.

### Substrate endothelialization

The bioreactor was placed under a wide-field Nikon-Ti microscope equipped with an incubation (Life Imaging Services, Switzerland) chamber maintaining a 37 °C and 5% CO_2_ environment. Prior to the insertion of the deformable PDMS membrane supporting a growth-arrested, differentiated endothelium, the bioreactor was sterilized by flushing all inner cavities with 70% ethanol. Luminal surfaces were then rinsed three times with PBS before filling the system with fresh medium. At this point the PDMS membrane supporting a layer of endothelial cells was mounted in the system under sterile conditions. The syringe pump providing the inflation was started and the inflation volume was ramped up until the required pressure generating the desired principal strain was reached. Afterwards, the desired flow rate was set and the cells were subjected to the flow for a minimum of 18 h. A PVDF flow sensor (PVDF Chemical Flowmeter 0.025–2.5 l/min, B.I.O-TECH e.K., Germany) was used to measure flow rate and fluctuation.

### Immunostaining

The following primary antibody was used: goat anti-VEC (Vascular Endothelial Cadherin; #6458) from Santa Cruz Biotechnology Inc. (USA). The secondary antibody was a Rabbit anti-Goat IgG (H + L) Superclonal Secondary Antibody, Alexa Fluor 488 (#A27012) conjugate from ThermoFisher Scientific (USA).

HUVECs were fixed and permeabilized for 10 min with 3% paraformaldehyde (PFA) and 0.1% Triton-X100 in PBS at room temperature (RT). The cells were then post-fixed with 3% PFA in PBS for 15 min. After washing the samples three times for 5 min with PBS, they were incubated with 5% bovine serum albumin (BSA) in PBS for 2 h at RT. The samples were incubated with TRITC-phalloidin (Sigma, USA) and goat anti-VEC primary antibody overnight at 4 °C. Subsequently, the samples were rinsed four times for 1 h each with 5% BSA in PBS and then were incubated with anti-goat-alexa-488 secondary antibody for 45 min at RT. Finally, the samples were washed three times (1 h each) in PBS, post-fixed for 2 min in 3% PFA, briefly washed again with PBS, mounted with Fluoroshield histology mounting medium (Sigma, USA) and imaged immediately.

### Image acquisition

The immunostained samples were imaged using an inverted Nikon-Ti spinning disk confocal microscope (Nikon, Japan) equipped with an Andor DU-888 camera (Oxford Instruments, United Kingdom) and a pE-100 LED illumination system (CoolLED Ltd, Andover, United Kingdom). Fluorescent images of immunostained HUVECs on the deformable PDMS membranes were acquired with a 20X, 0.75 NA air objective (Plan Apo, Nikon, Japan), using FITC, TRITC, and DAPI filters, respectively. Large images up to 6 mm × 6 mm were acquired by using the Custom Multipoint/Large Image function of NIS Elements (NIS Elements, Nikon, Japan) in combination with an autofocus routine followed by automated stitching.

### Image analysis

Images were analyzed and processed with Fiji (National Institutes of Health, USA). For the comparison of the CFD with the experimental results, cells in the selected regions were tracked manually and the “Measure” routine was used to determine the area, center of mass as well as the angle of the long axis of a fitted ellipse. The latter was used to generate a line plot showing the orientation of the tracked cells (with the line length scaled arbitrarily to the length of the fitted ellipse). Angles for the selected regions were plotted for comparison with the CFD predicted fluid direction angles in a rose plot using a binning of 15 degrees. Cell density was measured using the “Analyze Particles” plug-in of Fiji on the fluorescent channel for cell nuclei.

### Finite Element Model

A finite element model of the inflation system was created using ABAQUS (Abaqus 6.9-1, Dassault Systèmes). The model was 2D axial symmetric (x-y plane, x being the radial axis and y being the vertical axis). 8-node biquadratic axisymmetric quadrilateral, hybrid, linear pressure, reduced integration elements were selected for all computations. The constitutive model applied for the PDMS membrane was the one determined in our previous investigations on the multiaxial large strain mechanical behavior of the material^53^. The membrane edge was constrained in the vertical and radial direction, to simulate the boundary conditions applied in the real system. The pressure loading was applied on the bottom surface of the membrane.

### Digital image correlation

Measurement of the deformation field of the inflated membrane was achieved by digital image correlation performed using a VIC-3D^TM^ Micro set-up (Correlated Solutions Inc.). VIC-Snap^TM^ and VIC-3D^TM^ software were used for image acquisition and post processing. The system allows to reconstruct the 3D displacement and field from its interpolation of the corresponding deformation fields. A dedicated inflation system was realized for the strain measurements, identical (in geometry and boundary conditions of the inflated membrane) to the one used in the bioreactor.

Speckles were drawn on the membrane using an airbrush system (Harder & Steenbeck Evolution Silverline Solo, Germany). Different pressure levels were applied in this investigation (75, 220, 250, and 480 mbar) as well as membranes of different thicknesses (in the range 400–500 μm). Images were acquired with a frequency of 5 Hz.

Prior to the analysis, the region of interest for displacement reconstruction was defined corresponding to the part of the membrane which was inflated. The parameters that determine the grid spacing in the analysis were automatically assigned based on the image quality. Confidence margin, exhaustive search, and consistency threshold were also applied.

### Computational Fluid Dynamics model

The flow in the channel with flat and inflated membrane configurations was analyzed with a CFD model using ANSYS CFX. Sensitivity analyses (flow rate up to 1.2 l/min, WD up to 15%) by conducting a 2-way fluid-structure interaction analysis showed that the interaction between fluid forces and membrane deformations are negligible.

The structured mesh with 1.3 × 10^6^ hexahedral elements was developed in ICEM CFD. A previously performed convergence study proved that further mesh refinement does not improve accuracy. All mesh quality criteria required by ANSYS CFX were satisfied^58^.

For all cases the mesh resolution near the membrane surface was selected as such as that the nondimensional distance to the first near-membrane grid point


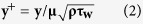


would match the recommended criterion


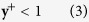


with y being the distance from the first cell center to the membrane, *μ* being the fluid viscosity, *ρ* the density, and *τ*_*W*_ being the WSS. Calculations were performed to quantify fluid-structure interaction effects and these were found negligible.

To reduce computational effort only one symmetric half of the channel was modeled. The fluid was modeled with a molar mass of 18.02 kg/kmol, a density of 993.3 kg/m^3^, and a dynamic viscosity of 6.913 × 10^−4^ kg m^−1^ s^−1^. This was considered to be a good approximation of the dynamic viscosity of the cell culture medium. As the flow rates from 0.1 l/min to 1.2 l/min result in Reynolds numbers of 563 to 7207, the flow regime exhibits transitional and turbulent regions^59^. As shown in Fig. 4A, for the flat membrane configuration, a laminar model could be applied up to 0.2 l/min. Due to the flow across the curved surface of the inflated membrane and the high range in Reynolds numbers, the two-equation eddy-viscosity Shear Stress Transport (SST) turbulence model^60^ was used for all other cases. The CFX solver accuracy was set to double precision and the root-mean-square values for mass conservation and velocity continuity were set to a target of 10^−5^.

### Micro Particle Image Velocimetry

Micro particle image velocimetry (μPIV) was performed using deionized water as working fluid, seeded with 3.55 μm fluorescent polystyrene particles (530/607 nm, microParticles GmbH). The experimental set-up consisted of an epi-fluorescence microscope system including a 3D stage system (LaVision FlowMaster Mitas) in combination with a 532 nm Nd:YAG laser (New Wave, Solo II-15) and a 2048 × 2048 pixel CCD camera (LaVision Imager ProX 4 M). For image acquisition a 5X, 0.16 NA microscope objective was employed (Zeiss EC “Plan-Neofluar”). For data post-processing the commercial software package Davis 7 (LaVision) was used. The volume flow of fluids was controlled with a peristaltic pump (P1500, Harvard Apparatus) and measured with a flow sensor (PVDF Chemical Flowmeter 0.025–2.5 l/min, B.I.O-TECH e.K., Germany). For each flow condition and measurement position 25 two-frame experimental images were acquired at a trigger rate of 4 Hz. The cross-correlation technique was used for analysis. The final interrogation window of 128 × 128 pixels overlapped by 50%, the corresponding vector spacing was 62.5 μm. The depth of correlation was determined to be about 124 μm, following ref. 61. After processing, the 25 experimental images were time-averaged. Further details on the experimental set-up have been previously reported in ref. 62.

### Statistical analysis

Statistical comparison of cell area for the 3 distinct regions analyzed in Fig. 7 was performed using a non-parametric Mann-Whitney test (α < 0.05). At least 3 independent experiments were analyzed for each of the respective regions. All quantitative measurements are represented as average ± the standard error of the mean.

## Additional Information

**How to cite this article**: Bachmann, B. J. *et al*. A Novel Bioreactor System for the Assessment of Endothelialization on Deformable Surfaces. *Sci. Rep.* **6**, 38861; doi: 10.1038/srep38861 (2016).

**Publisher's note:** Springer Nature remains neutral with regard to jurisdictional claims in published maps and institutional affiliations.

## Supplementary Material

Supplementary Information

Supplementary Video 1

Supplementary Video 2

Supplementary Video 3

Supplementary Video 4

## References

[b1] Mozaffarian D. . On behalf of the American Heart Association Statistics Committee and Stroke Statistics Subcommittee. Heart disease and stroke statistics - 2016 update: a report from the American Heart Association. Circulation 133, e38–e360 (2016). 26673558 10.1161/CIR.0000000000000350

[b2] Ziaeian B. & Fonarow G. C. Epidemiology and aetiology of heart failure. Nat Rev Cardiol 13, 368–378 (2016). 26935038 10.1038/nrcardio.2016.25 PMC4868779

[b3] Wilhelm M. J., Ruschitzka F. & Falk V. Destination therapy-time for a paradigm change in heart failure therapy. Swiss Med. Wkly. 143, 1–13 (2013). 10.4414/smw.2013.13729 23572384

[b4] Dunlay S. M. & Roger V. L. Understanding the Epidemic of Heart Failure: Past, Present, and Future. Curr. Heart Fail. Rep. 11, 404–415 (2014). 25182014 10.1007/s11897-014-0220-x PMC4224604

[b5] Silva Enciso J. Mechanical Circulatory Support: Current Status and Future Directions. Prog. Cardiovasc. Dis. 58, 444–454 (2016). 26780234 10.1016/j.pcad.2016.01.006

[b6] Jaffer I. H., Fredenburgh J. C., Hirsh J. & Weitz J. I. Medical device-induced thrombosis: What causes it and how can we prevent it? J. Thromb. Haemost. 13, S72–S81 (2015). 26149053 10.1111/jth.12961

[b7] McGuigan A. P. & Sefton M. V. The influence of biomaterials on endothelial cell thrombogenicity. Biomaterials 28, 2547–2571 (2007). 17316788 10.1016/j.biomaterials.2007.01.039 PMC1868518

[b8] Susen S., Rauch A., Van Belle E., Vincentelli A. & Lenting P. J. Circulatory support devices: Fundamental aspects and clinical management of bleeding and thrombosis. J. Thromb. Haemost. 13, 1757–1767 (2015). 26302994 10.1111/jth.13120

[b9] Baiguera S. & Ribatti D. Endothelialization approaches for viable engineered tissues. Angiogenesis 16, 1–14 (2013). 23010872 10.1007/s10456-012-9307-8

[b10] Liu X. . Blood compatible materials: State of the art. J. Mater. Chem. B 2, 5718–5738 (2014). 10.1039/c4tb00881b 32262016

[b11] Franco D. . Control of initial endothelial spreading by topographic activation of focal adhesion kinase. Soft Matter 7, 7313 (2011).

[b12] Liliensiek S. J. . Modulation of human vascular endothelial cell behaviors by nanotopographic cues. Biomaterials 31, 5418–5426 (2010). 20400175 10.1016/j.biomaterials.2010.03.045 PMC2907086

[b13] Morgan J. P. . Formation of microvascular networks *in vitro*. Nat. Protoc. 8, 1820–36 (2013). 23989676 10.1038/nprot.2013.110

[b14] Pang J. H. . *In situ* Endothelialization: Bioengineering Considerations to Translation. Small 11, 6248–6264 (2015). 26460851 10.1002/smll.201402579

[b15] Li S. & Henry J. J. D. Nonthrombogenic approaches to cardiovascular bioengineering. Annu. Rev. Biomed. Eng. 13, 451–75 (2011). 21639778 10.1146/annurev-bioeng-071910-124733

[b16] Liu T., Liu S., Zhang K., Chen J. & Huang N. Endothelialization of implanted cardiovascular biomaterial surfaces: The development from *in vitro* to *in vivo*. J. Biomed. Mater. Res. - Part A 102, 3754–3772 (2014). 10.1002/jbm.a.35025 24243819

[b17] Pennel Z., Zilla P. & Bezuidenhout D. Biomaterials in Vascular Graft Surgery in Reference Module in Materials Science and Materials Engineering, doi: 10.1016/B978-0-12-803581-8.02159-7 (Elsevier, 2016).

[b18] Ren X. . Surface modification and endothelialization of biomaterials as potential scaffolds for vascular tissue engineering applications. Chemical Society Reviews 44, 5680–5742 (2015). 26023741 10.1039/c4cs00483c

[b19] Kohn J. C. . Cooperative effects of matrix stiffness and fluid shear stress on endothelial cell behavior. Biophys. J. 108, 471–478 (2015). 25650915 10.1016/j.bpj.2014.12.023 PMC4317546

[b20] Dolan J. M., Meng H., Sim F. J. & Kolega J. Differential gene expression by endothelial cells under positive and negative streamwise gradients of high wall shear stress. Am. J. Physiol. Cell Physiol. 305, C854–66 (2013). 23885059 10.1152/ajpcell.00315.2012 PMC3798684

[b21] Dolan J. M., Meng H., Singh S., Paluch R. & Kolega J. High fluid shear stress and spatial shear stress gradients affect endothelial proliferation, survival, and alignment. Ann. Biomed. Eng. 39, 1620–1631 (2011). 21312062 10.1007/s10439-011-0267-8 PMC4809045

[b22] Huang L. & Helmke B. P. Polarized Actin Structural Dynamics in Response to Cyclic Uniaxial Stretch. Cell. Mol. Bioeng. 8, 160–177 (2015). 25821527 10.1007/s12195-014-0370-7 PMC4372154

[b23] Franco D. . Accelerated endothelial wound healing on microstructured substrates under flow. Biomaterials 34, 1488–97 (2013). 23182348 10.1016/j.biomaterials.2012.10.007

[b24] Milde F. . Cell Image Velocimetry (CIV): boosting the automated quantification of cell migration in wound healing assays. Integr. Biol. (Camb). 4, 1437–47 (2012). 23047374 10.1039/c2ib20113e

[b25] Orsenigo F. . Phosphorylation of VE-cadherin is modulated by haemodynamic forces and contributes to the regulation of vascular permeability *in vivo*. Nat. Commun. 3, 1208 (2012). 23169049 10.1038/ncomms2199 PMC3514492

[b26] Potthoff E. . Toward a rational design of surface textures promoting endothelialization. Nano Lett. 14, 1069–79 (2014). 24428164 10.1021/nl4047398

[b27] Pakravan H. A., Saidi M. S. & Firoozabadi B. A mechanical model for morphological response of endothelial cells under combined wall shear stress and cyclic stretch loadings. Biomech. Model. Mechanobiol. doi: 10.1007/s10237-015-0756-z (2016). 26769119

[b28] Estrada R. . Endothelial Cell Culture Model for Replication of Physiological Profiles. Anal. Chem. 3170–3177 (2011). 21413699 10.1021/ac2002998

[b29] Tamiello C., Buskermolen A. B. C., Baaijens F. P. T., Broers J. L. V. & Bouten C. V. C. Heading in the Right Direction: Understanding Cellular Orientation Responses to Complex Biophysical Environments. Cell. Mol. Bioeng. 9, 12–37 (2015). 26900408 10.1007/s12195-015-0422-7 PMC4746215

[b30] Jufri N. F., Mohamedali A., Avolio A. & Baker M. S. Mechanical stretch: physiological and pathological implications for human vascular endothelial cells. Vasc. Cell 7, 8 (2015). 26388991 10.1186/s13221-015-0033-z PMC4575492

[b31] Sinha R. . A medium throughput device to study the effects of combinations of surface strains and fluid-flow shear stresses on cells. Lab Chip 15, 429–39 (2014). 10.1039/c4lc01259c 25377548

[b32] Zheng W. . A microfluidic flow-stretch chip for investigating blood vessel biomechanics. Lab Chip 12, 3441–50 (2012). 22820518 10.1039/c2lc40173h

[b33] Tsukurov O. I. . The Response of Adult Human Saphenous Vein Endothelial Cells to Combined Pressurized Pulsatile Flow and Cyclic Strain, *In Vitro*. Ann. Vasc. Surg. 14, 260–267 (2000). 10796958 10.1007/s100169910044

[b34] Benbrahim A. . A compliant tubular device to study the influences of wall strain and fluid shear stress on cells of the vascular wall. J. Vasc. Surg. 20, 184–194 (1994). 8040941 10.1016/0741-5214(94)90005-1

[b35] Zhao S. . Synergistic Effects of Fluid Shear Stress and Cyclic Circumferential Stretch on Vascular Endothelial Cell Morphology and Cytoskeleton. Arterioscler. Thromb. Vasc. Biol. 15, 1781–1786 (1995). 7583556 10.1161/01.atv.15.10.1781

[b36] Moore J. E. . A device for subjecting vascular endothelial cells to both fluid shear stress and circumferential cyclic stretch. Ann. Biomed. Eng. 22, 416–422 (1994). 7998687 10.1007/BF02368248

[b37] Al-Azawy M. G., Turan A. & Revell A. Assessment of turbulence models for pulsatile flow inside a heart pump. Comput. Methods Biomech. Biomed. Engin. 13, 271–285 (2016). 10.1080/10255842.2015.1015527 25816074

[b38] Sonntag S. J. . Simulation of a pulsatile total artificial heart: Development of a partitioned Fluid Structure Interaction model. J. Fluids Struct. 38, 187–204 (2013).

[b39] Wittek A. . Cyclic three-dimensional wall motion of the human ascending and abdominal aorta characterized by time-resolved three-dimensional ultrasound speckle tracking. Biomech. Model. Mechanobiol. 15, 1–14 (2016). 10.1007/s10237-016-0769-2 26897533

[b40] Kroll M. H., Hellums J. D., McIntire L. V., Schafer A. I. & Moake J. L. Platelets and shear stress. Blood 88, 1525 LP-1541 (1996). 8781407

[b41] Amaya R., Pierides A. & Tarbell J. M. The Interaction between Fluid Wall Shear Stress and Solid Circumferential Strain Affects Endothelial Gene Expression. PLoS One 10, 1–18 (2015). 10.1371/journal.pone.0129952 PMC4492743 26147292

[b42] Dancu M. B. & Tarbell J. M. Large Negative Stress Phase Angle (SPA) attenuates nitric oxide production in bovine aortic endothelial cells. J. Biomech. Eng. 128, 329–34 (2006). 16706582 10.1115/1.1824120

[b43] Stefopoulos G., Robotti F., Falk V., Poulikakos D. & Ferrari A. Endothelialization of Rationally Microtextured Surfaces with Minimal Cell Seeding Under Flow. Small 1–14, doi: 10.1002/smll.201503959 (2016). 27346806

[b44] Bartoli C. R. . Inhibition of ADAMTS-13 by Doxycycline Reduces von Willebrand Factor Degradation During Supraphysiological Shear Stress: Therapeutic Implications for Left Ventricular Assist Device-Associated Bleeding. JACC Hear. Fail. 3, 860–869 (2015). 10.1016/j.jchf.2015.06.016 26454844

[b45] Robotti F. . The influence of surface micro-structure on endothelialization under supraphysiological wall shear stress. Biomaterials 35, 8479–86 (2014). 25017097 10.1016/j.biomaterials.2014.06.046

[b46] Niklason L. E. . Functional arteries grown *in vitro*. Science 284, 489–493 (1999). 10205057 10.1126/science.284.5413.489

[b47] Fry D. L. Certain histological and chemical responses of the vascular interface to acutely induced mechanical stress in the aorta of the dog. Circ. Res. 24, 93–108 (1969). 5763742 10.1161/01.res.24.1.93

[b48] Fry D. L. Certain Chemorheologic Considerations Regarding the Blood Vascular Interface with Particular Reference to Coronary Artery Disease. Circulation 40, IV-38 LP-IV-57 (1969).

[b49] Kane R. S., Takayama S., Ostuni E., Ingber D. E. & Whitesides G. M. Patterning proteins and cells using soft lithography. Biomaterials 20, 2363–2376 (1999). 10614942 10.1016/s0142-9612(99)00165-9

[b50] Singhvi R. . Engineering cell shape and function. Science. 264, 696 LP-698 (1994). 10.1126/science.8171320 8171320

[b51] Ren X. . Surface modification and endothelialization of biomaterials as potential scaffolds for vascular tissue engineering applications. Chem. Soc. Rev. 44, 5680–5742 (2015). 26023741 10.1039/c4cs00483c

[b52] Martinez-Duarte R. Easy and inexpensive fabrication of PDMS films of different thicknesses. Chips and Tips (Lab on a Chip) (2012). Available at: http://blogs.rsc.org/chipsandtips/2012/04/18/easy-and-inexpensive-fabrication-of-pdms-films-of-different-thicknesses. (Accessed: 15th June 2016).

[b53] Hopf R. . Experimental and theoretical analyses of the age-dependent large-strain behavior of Sylgard 184 (10:1) silicone elastomer. J. Mech. Behav. Biomed. Mater. 60, 425–437 (2016). 26990071 10.1016/j.jmbbm.2016.02.022

[b54] Oladipupo S. . VEGF is essential for hypoxia-inducible factor-mediated neovascularization but dispensable for endothelial sprouting. Proc. Natl. Acad. Sci. USA 108, 13264–13269 (2011). 21784979 10.1073/pnas.1101321108 PMC3156154

[b55] Murakami. Fibroblast growth factor regulation of neovascularization. Curr Opin Hematol 15, 215–220 (2008). 18391788 10.1097/MOH.0b013e3282f97d98 PMC2745288

[b56] Lampugnani M. G. . The molecular organization of endothelial cell to cell junctions: differential association of plakoglobin, beta-catenin, and alpha-catenin with vascular endothelial cadherin (VE-cadherin). J. Cell Biol. 129, 203–17 (1995). 7698986 10.1083/jcb.129.1.203 PMC2120375

[b57] Lampugnani M. G. . Cell confluence regulates tyrosine phosphorylation of adherens junction components in endothelial cells. J. Cell Sci. 110 (Pt 1) 2065–77 (1997). 9378757 10.1242/jcs.110.17.2065

[b58] ANSYS Inc. ANSYS® Academic Research. ANSYS CFX-Solver Modeling Guide 15317, 448–451 (2013).

[b59] Patel V. C. & Head M. R. Some observations on skin friction and velocity profiles in fully developed pipe and channel flows. J. Fluid Mech. 38, 181 (1969).

[b60] Menter F. R. Two-equation eddy-viscosity turbulence models for engineering applications. AIAA J. 32, 1598–1605 (1994).

[b61] Olsen M. G. & Adrian R. J. Out-of-focus effects on particle image visibility and correlation in microscopic particle image velocimetry. Exp. Fluids 29, (2000).

[b62] Marschewski J. . Mixing with herringbone-inspired microstructures: overcoming the diffusion limit in co-laminar microfluidic devices. Lab Chip 15, 1923–1933 (2015). 25737365 10.1039/c5lc00045a

