# Supplementary material for: A Novel Bioreactor System for the Assessment of Endothelialization on Deformable Surfaces
Source: Sci Rep. 2016 Dec 12;6:38861. doi: 10.1038/srep38861 (PMC5150819; doi:10.1038/srep38861)
Supplement: Supplementary Information [file srep38861-s1.pdf]

Supplementary Material

A Novel Bioreactor System for the Assessment of Endothelialization on Deformable Surfaces

Björn Bachmann [a§], Laura Bernardi [b§], Christian Loosli [c§], Julian Marschewski [a], Michela Perrini [b], Martin Ehrbar [d], Paolo Ermanni [c], Dimos Poulikakos [a], Aldo Ferrari [a*], and Edoardo Mazza [b].*

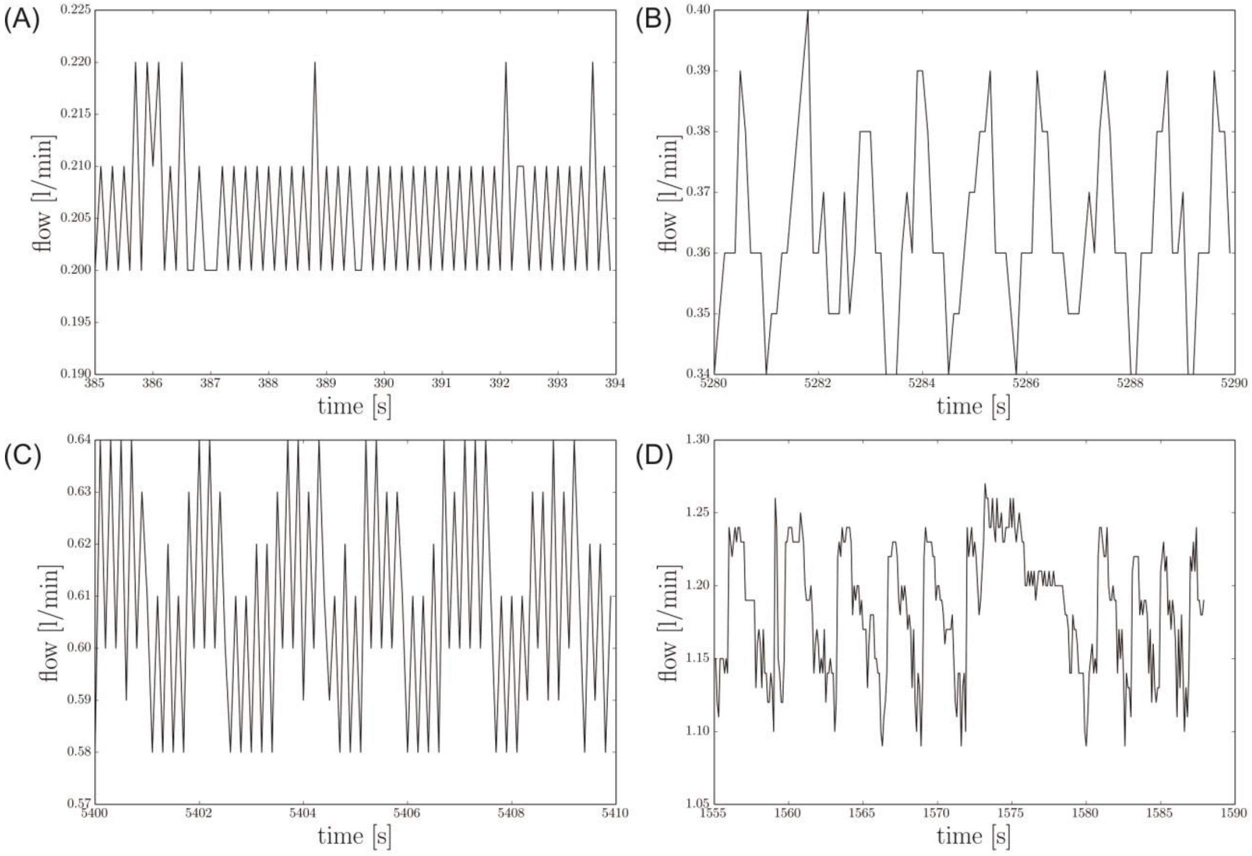

Supplementary Figure 1: Detection of flow rate fluctuation in the reactor system. Measurements were acquired after the fluid dampener and before the reactor system for flow rates of (**A**) 0.2 l/min, (**B**) 0.4 l/min, (**C**) 0.6 l/min and (**D**) 1.2 l/min, respectively.

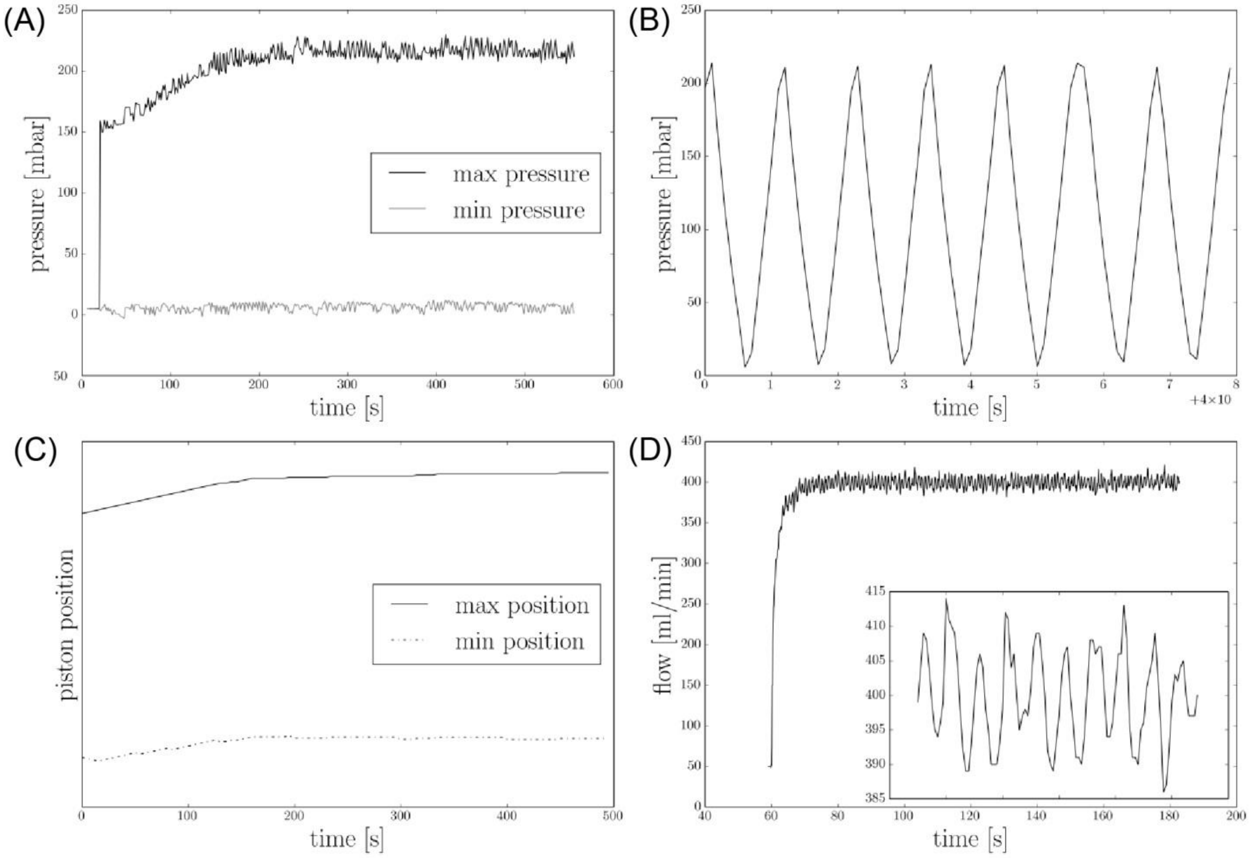

Supplementary Figure 2. Control signals (**A**): Pressure max and min signals. At the beginning of the experiment the pressure amplitude is gradually increased until it reaches the target value (220 mbar) and is subsequently kept constant for the remainder of the test. (**B**): Particular of the pressure signal. The pressure cycles between 0 mbar and the target pressure (220 mbar). (**C**): Piston positions. The position of the piston is changing in order to keep the pressure constant. (**D**): Flow signal. The flow fluctuates within about 10% of the mean value.

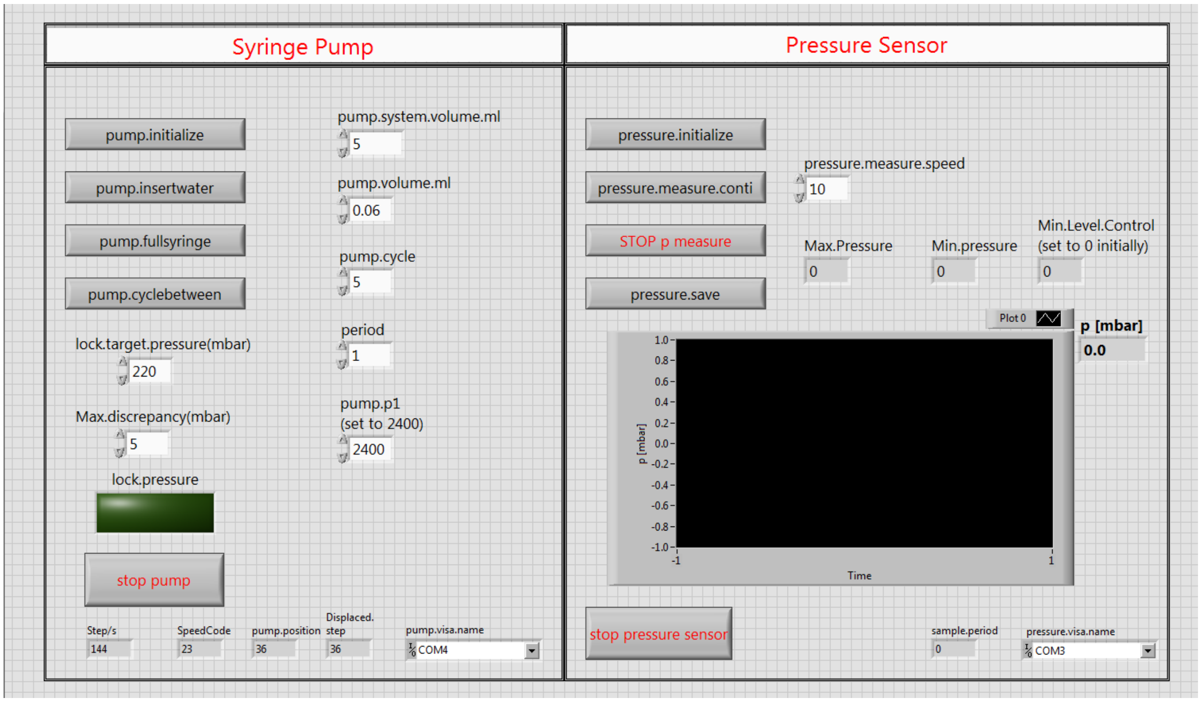

Supplementary Figure 3: LabVIEW control interface. Left: syringe pump control. The pump is first initialized (pump.initialize), then the cylinder circuit is filled with PBS (pump.insertwater) and the piston moves to the initial position (pump.fullsyringe). When the "pump.cyclebetween" and the "lock.pressure" buttons are pressed, the piston cycles between the "pump.p1" and the "Min.Level.Control" (right side) positions. These two positions are controlled in order to keep the pressure constant. The target pressure is set in the "lock.target.pressure (mbar)" control, while the max discrepancy is set in the "max.discrepancy" control. The period can be adjusted varying the "period" entry. Right: pressure sensor control. The sensor is initialized (pressure.initialize) and measured (pressure.measure.conti). The sampling rate can be set via the "pressure.measure.speed" box. The pressure signal is displayed in the plot.

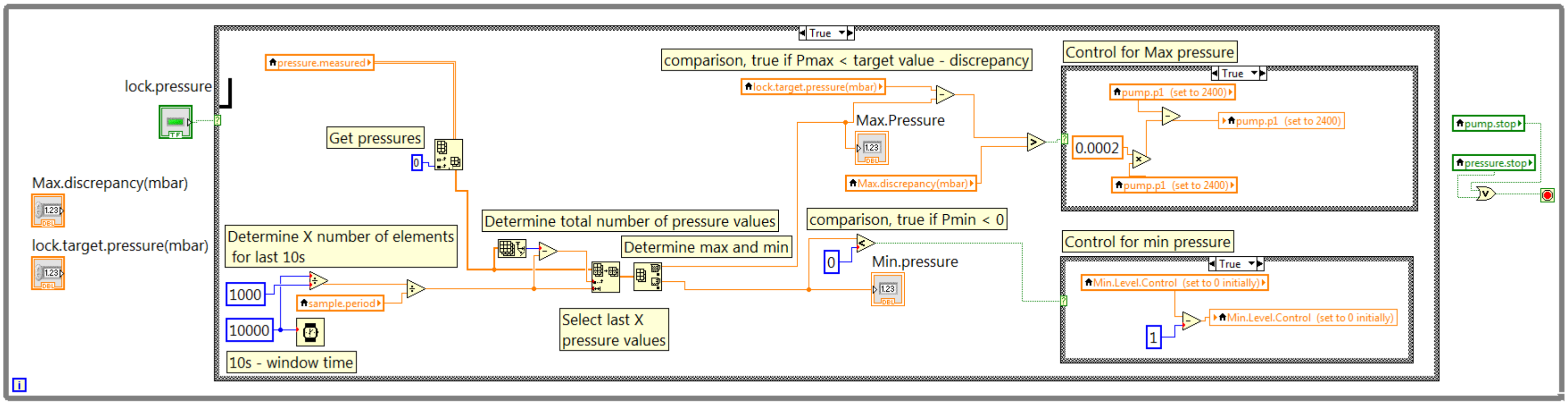

Supplementary Figure 4: Pressure control loop. The pressure signal is registered and every 10 s the max and min values are compared to the target value (max pressure) and the min pressure (0 mbar). In particular, if $\mathbf{Max.Pressure} < \mathbf{lock.target.pressure} - \mathbf{discrepancy}$, the pump.p1 piston position is decreased, and more volume is injected into the circuit, increasing the pressure. When the $\mathbf{Min.pressure} < \mathbf{0}$, the Min.Level.Control piston position is changed in order to increase the minimum pressure again.

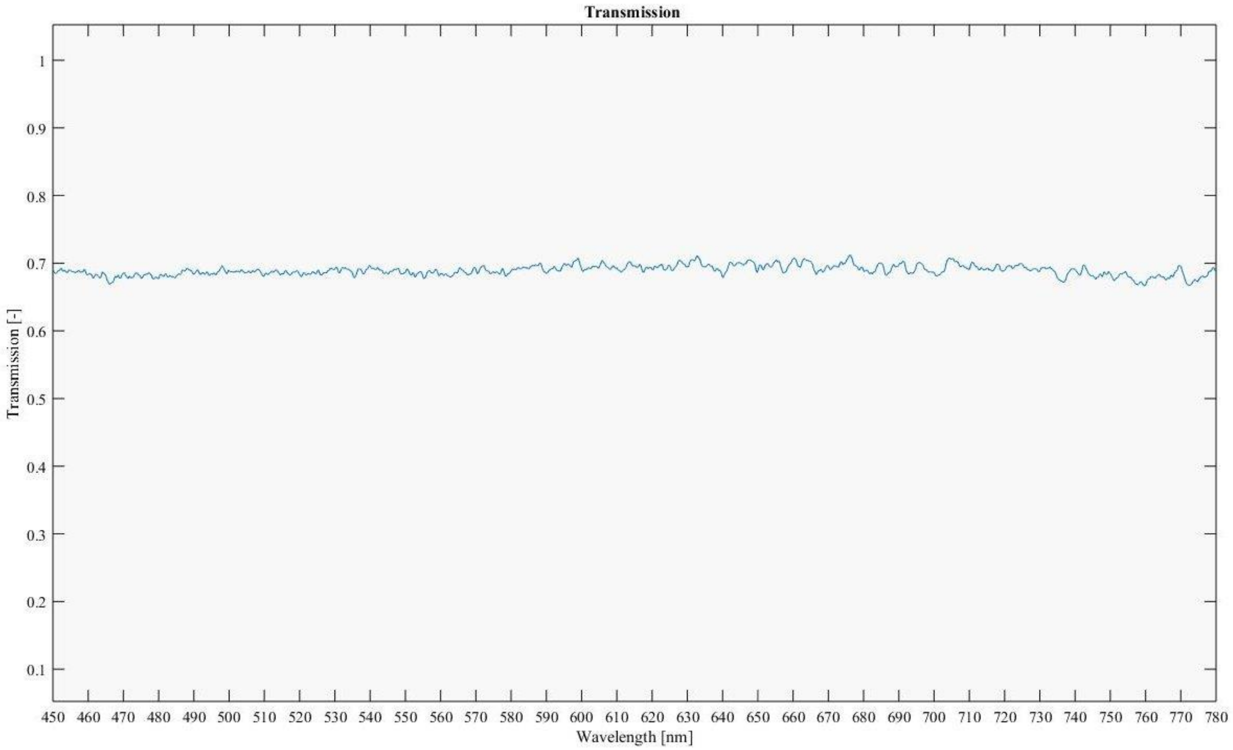

Supplementary Figure 5: Optical conductivity. The transmission efficiency of a reactor mounted with a PDMS membrane and filled with PBS was measured with a white-light spectrometer. The average transmission across wavelengths from 450 nm to 780 nm was 68.9 %.

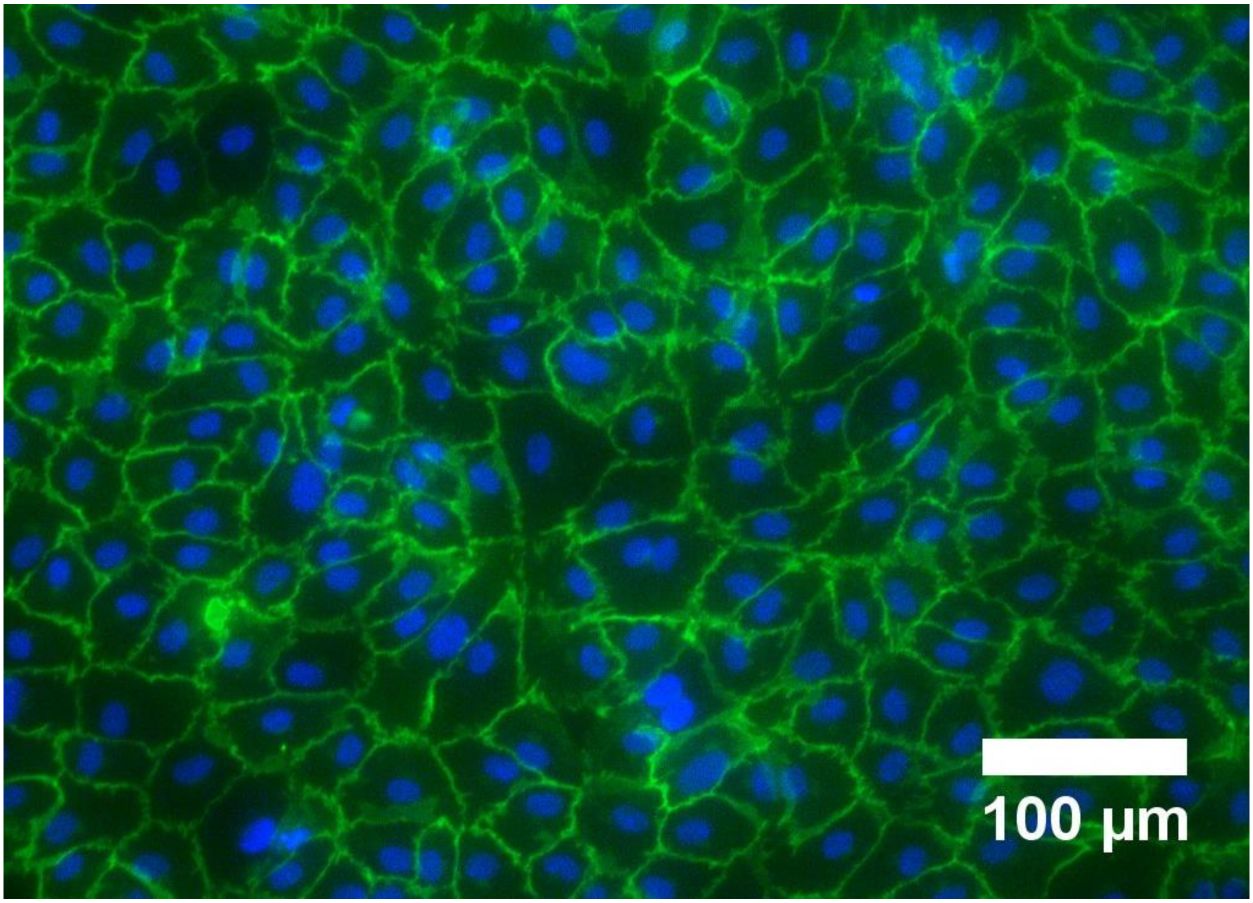

Supplementary Figure 6: Immunostaining of HUVECs grown under static conditions 3 days after seeding. VE-Cadherin (VEC) is visualized in the green channel and nuclei are visualized in the blue channel. Well-defined VEC junctions are indicative of a fully developed monolayer.

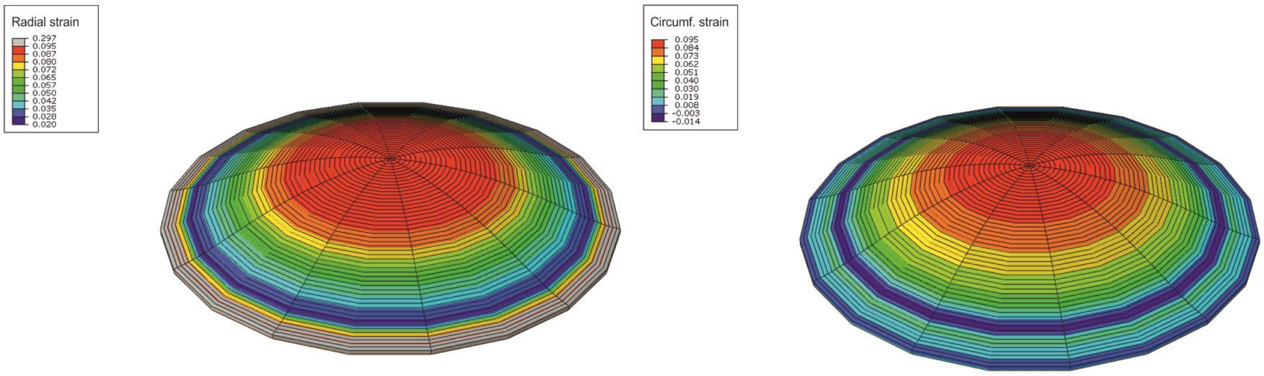

Supplementary Figure 7: Radial (left) and circumferential (right) strain for a 400 μm membrane loaded with 220 mbar pressure. Circumferential and radial strain values are similar in the central region. The state of membrane deformation is considered equibiaxial for a region in which the difference between strain components is less than 10% of their respective values. For the case represented in the figure it correspond to 10% of the membrane area.

Supplementary Figures 8-11: High resolution images of actin staining of HUVECs covered membranes after exposure to various flowrates for 18 h (corresponding to the substrates displayed in Figure 5 of the manuscript). The flow direction is indicated by the white arrow.

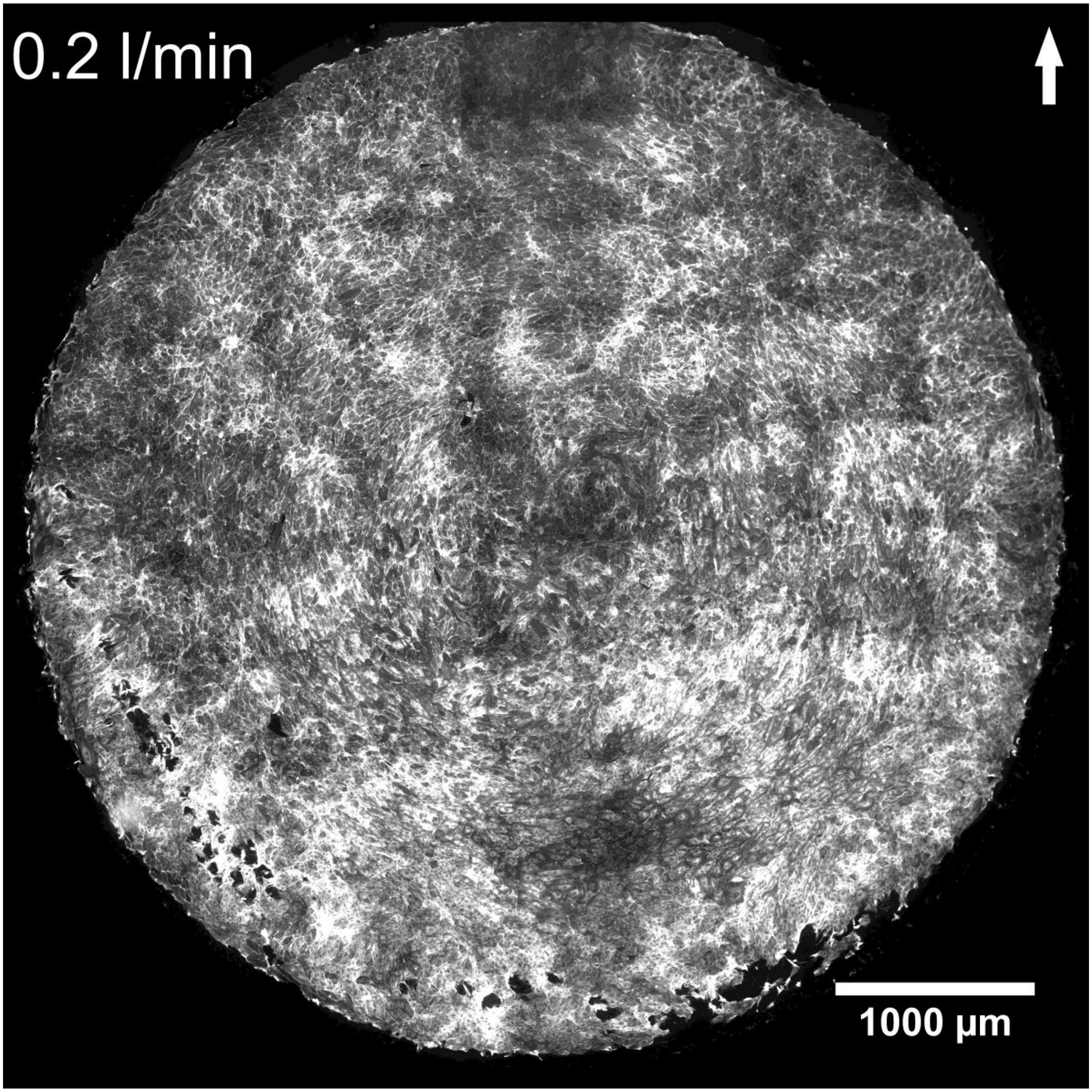
0.2 l/min
1000 µm

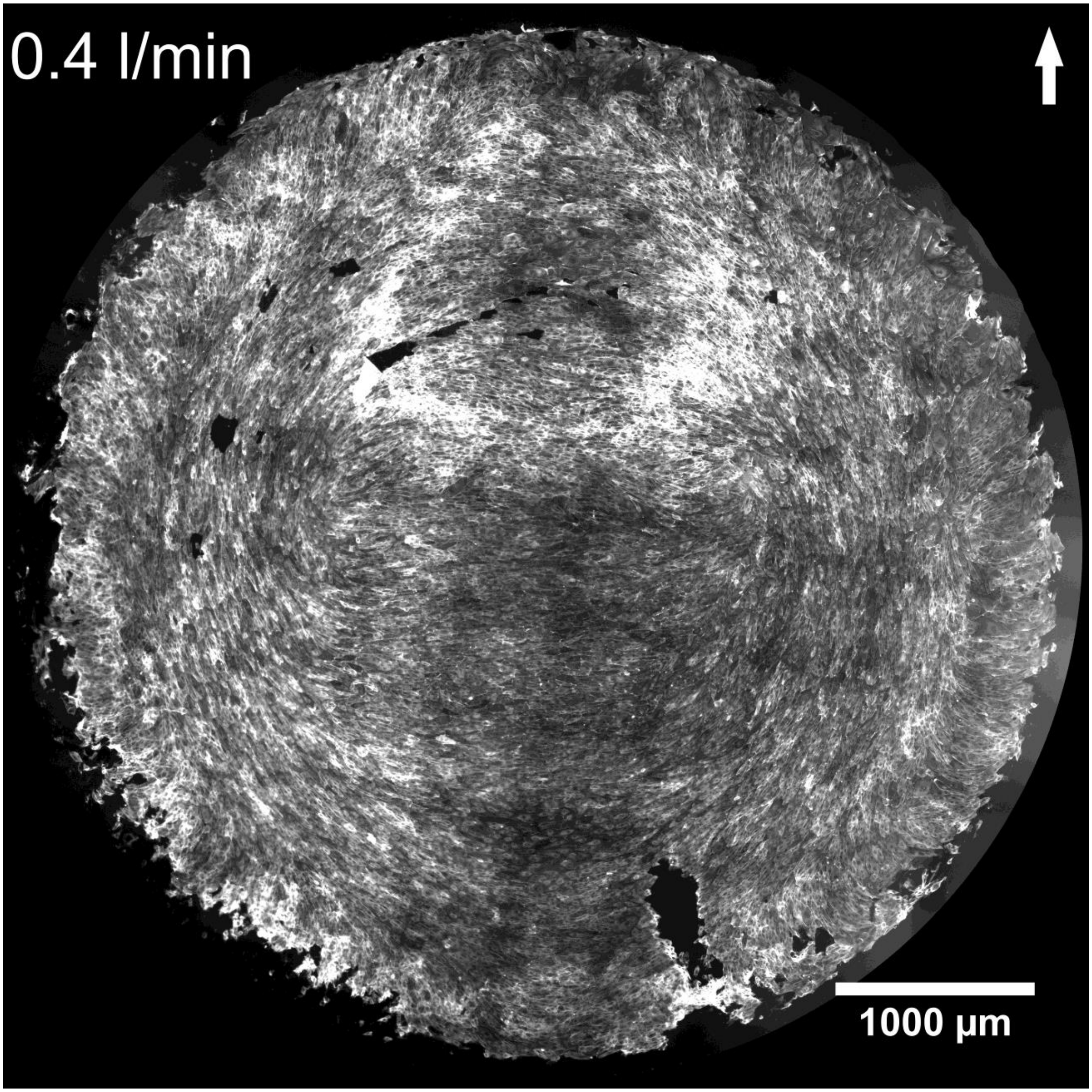
0.4 l/min
1000 µm

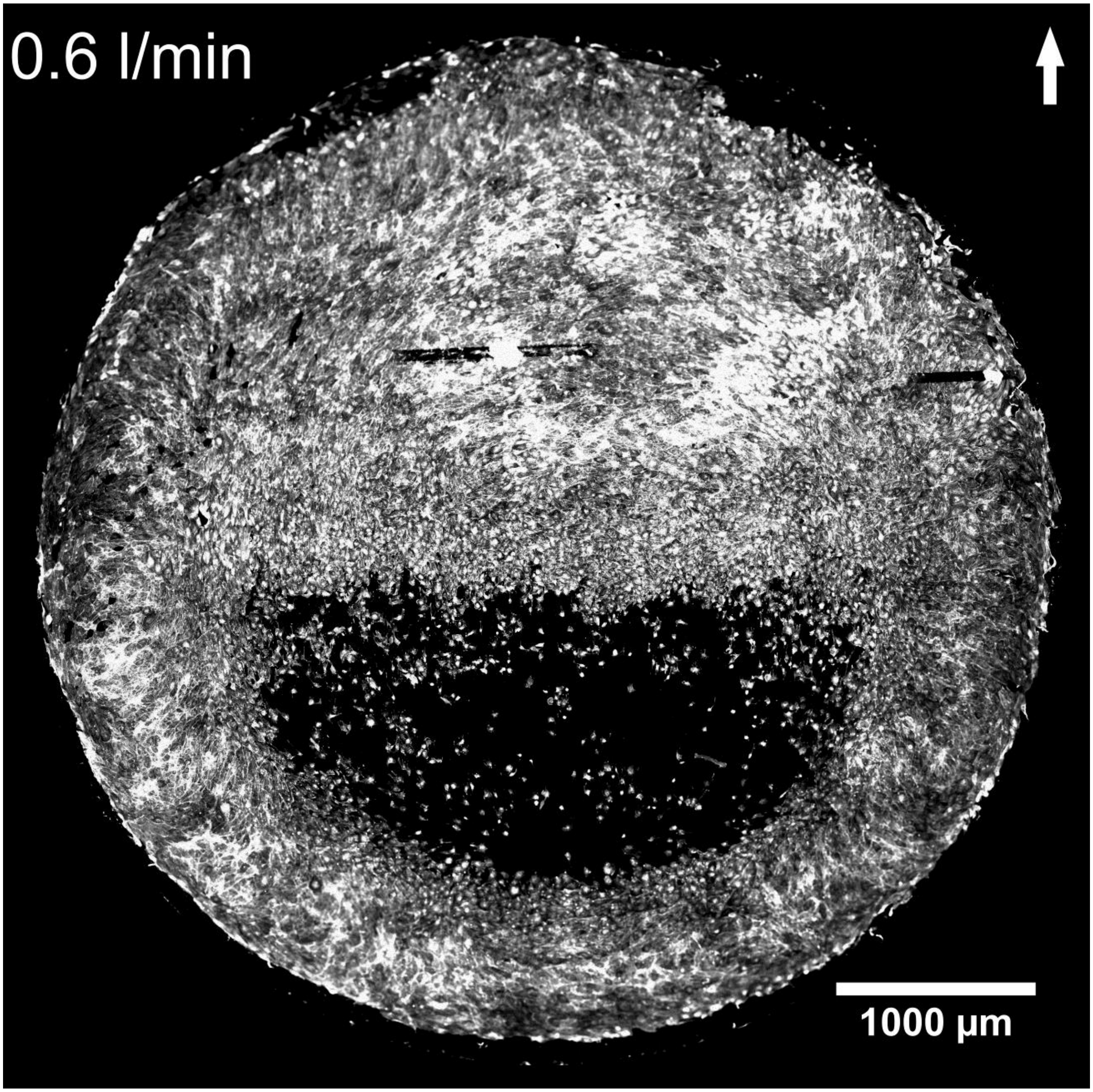

0.6 l/min
1000 µm

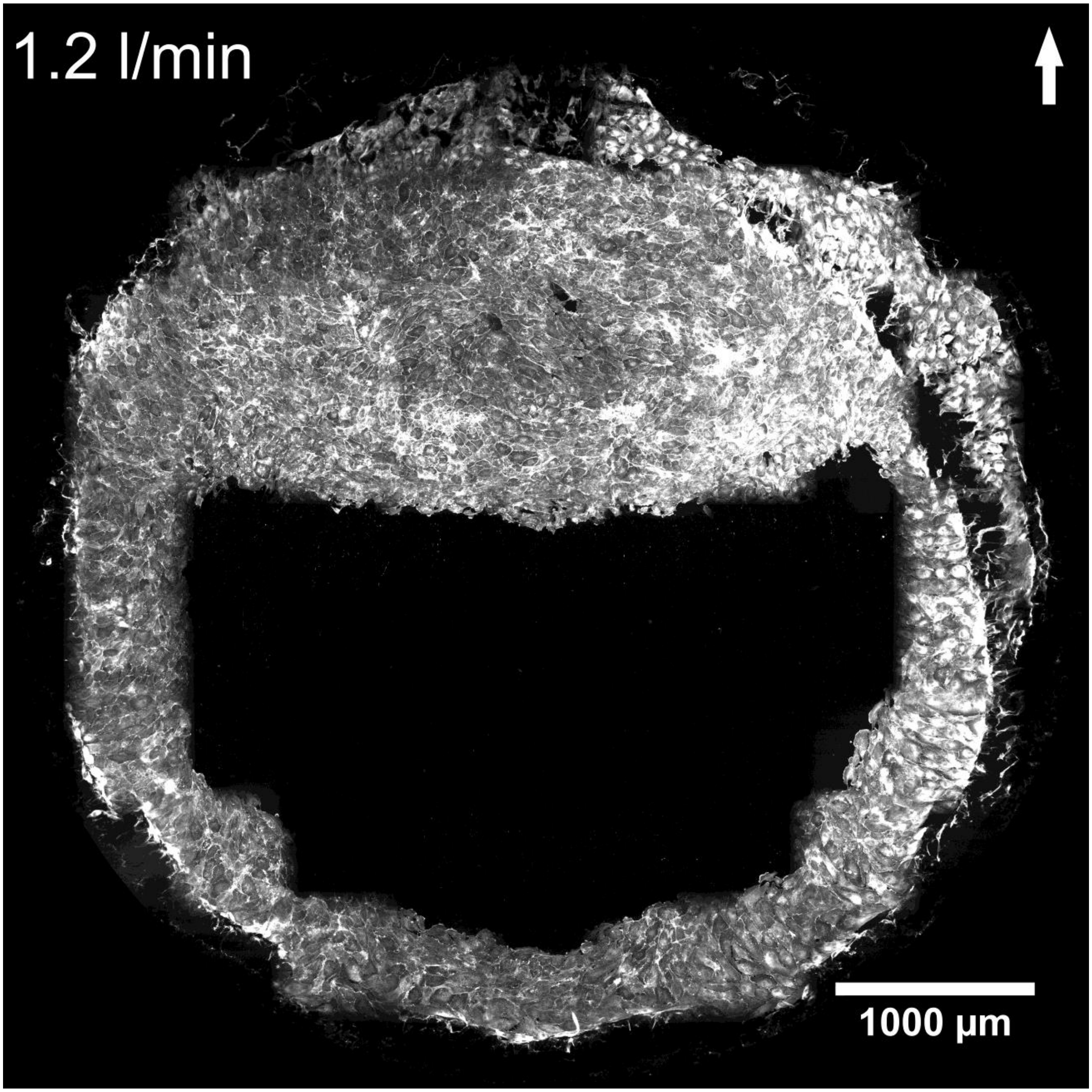
1.2 l/min
1000 µm

Supplementary Figures 12-14: High resolution images corresponding to region I defined and displayed in Figure 8 of the manuscript.

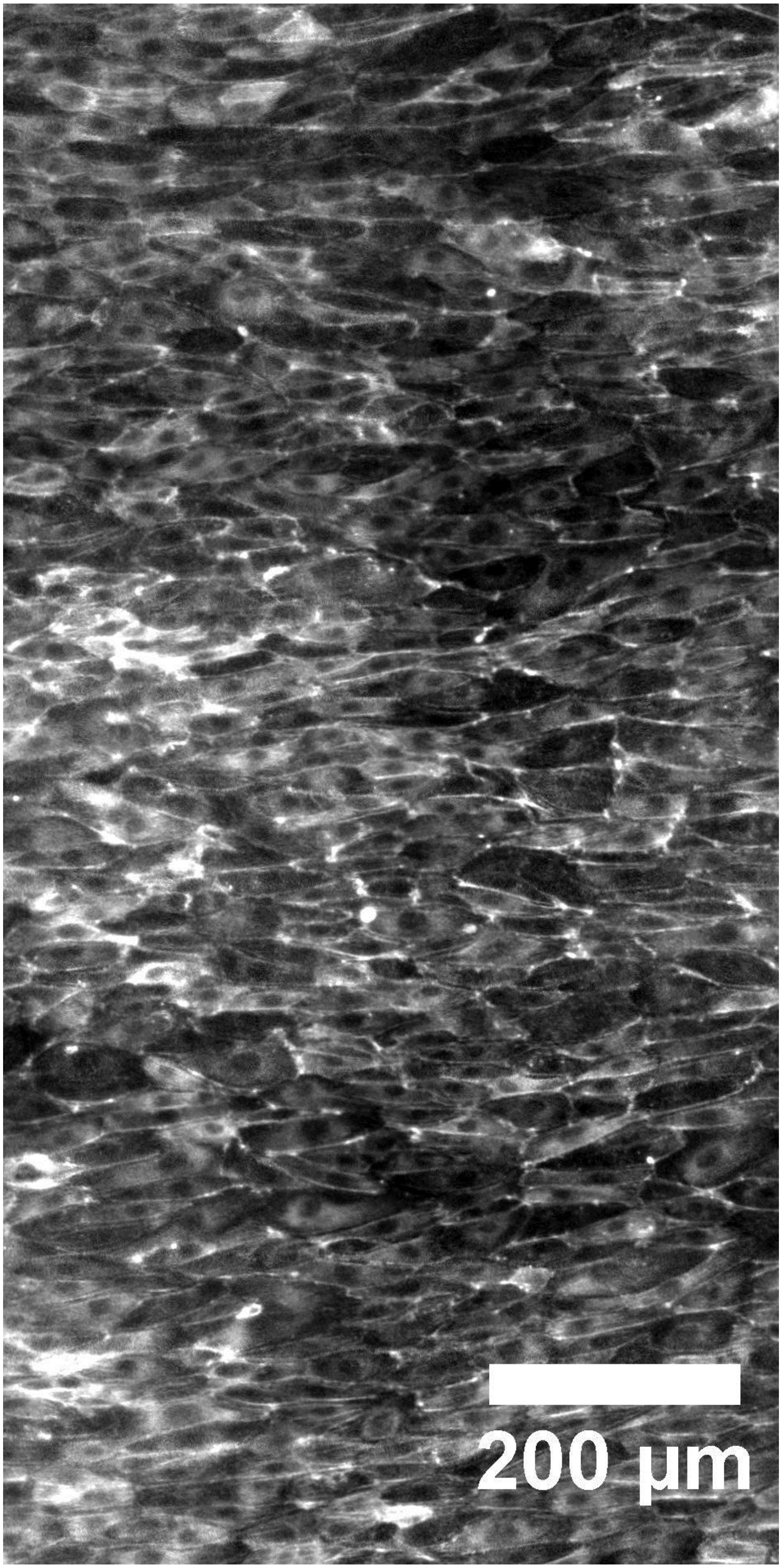
200 µm

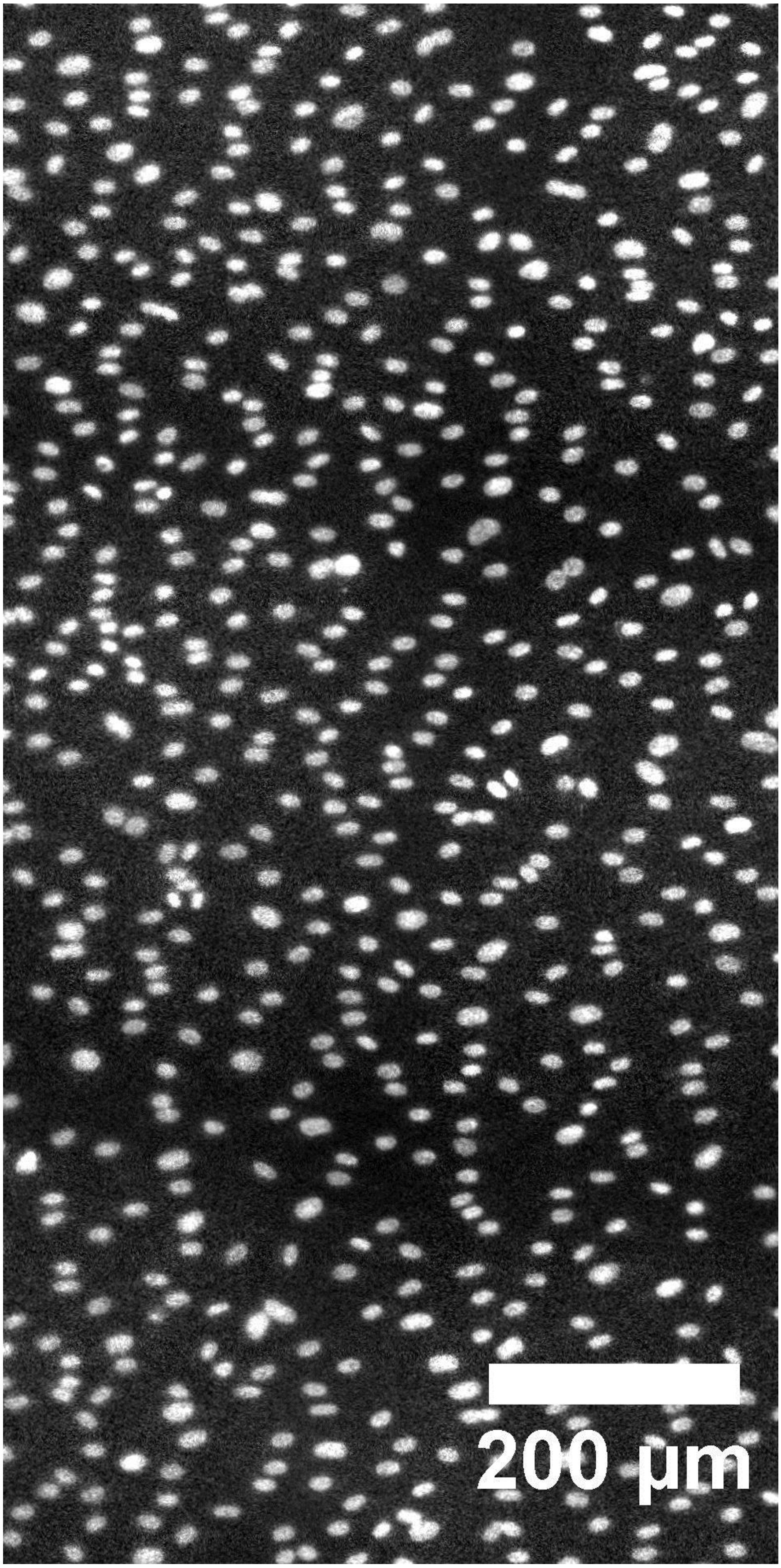
200 µm

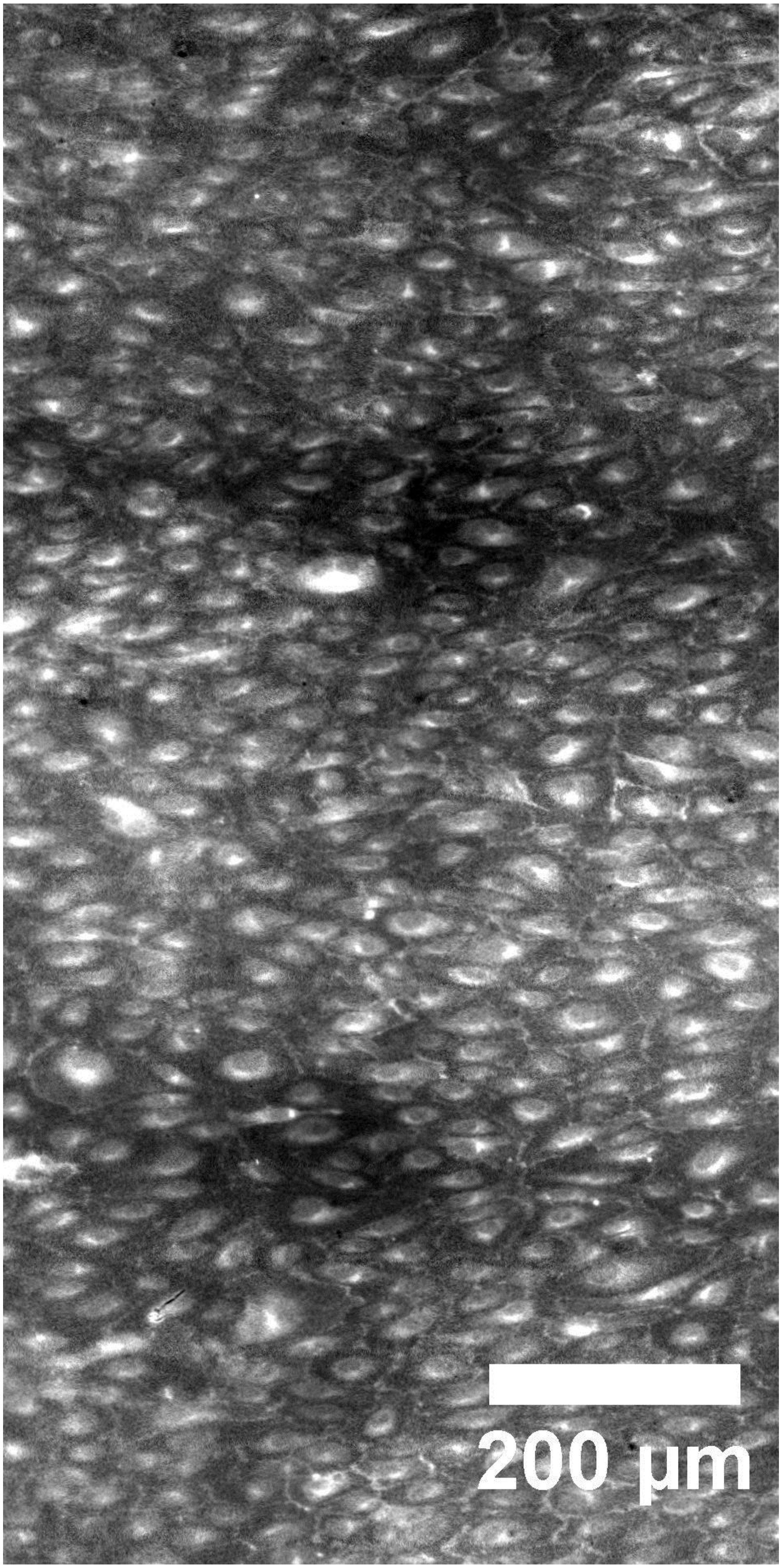
200 µm

Supplementary Figures 15-17: High resolution images corresponding to region II defined and displayed in Figure 8 of the manuscript.

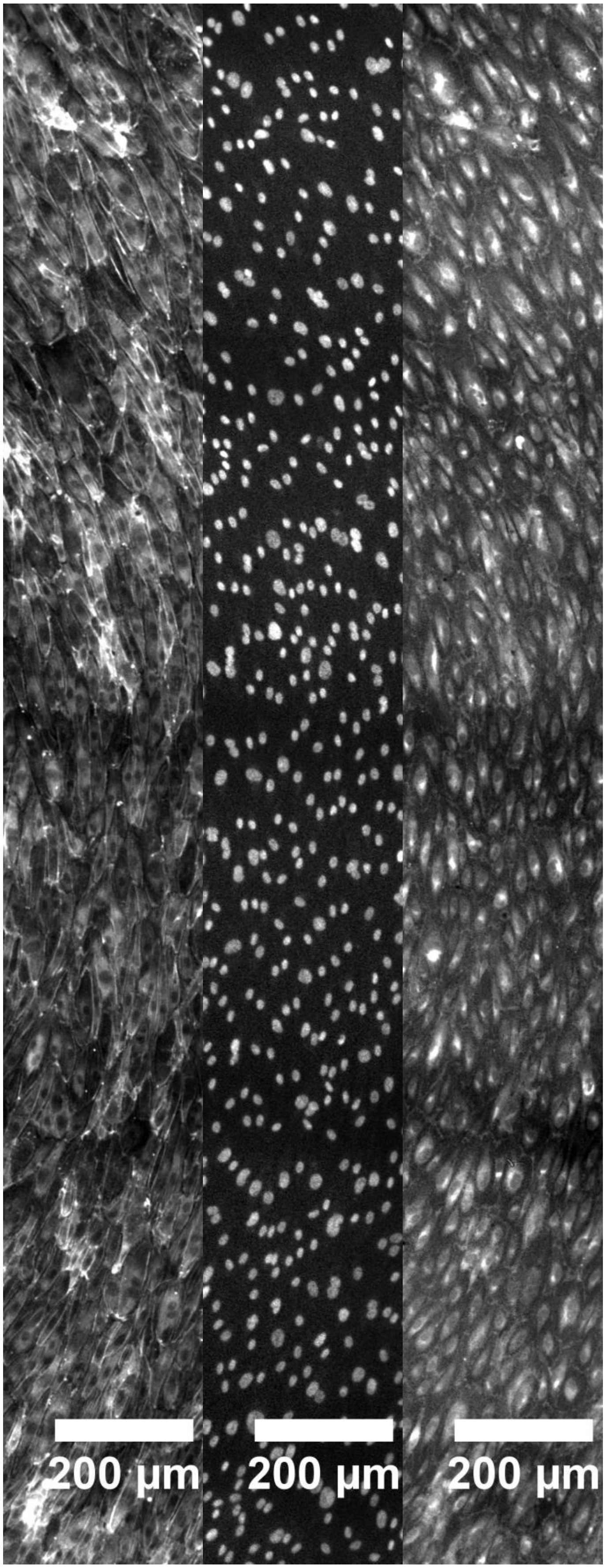
200 µm
200 µm
200 µm

Supplementary Figures 18-20: High resolution images corresponding to region III defined and displayed in Figure 8 of the manuscript.

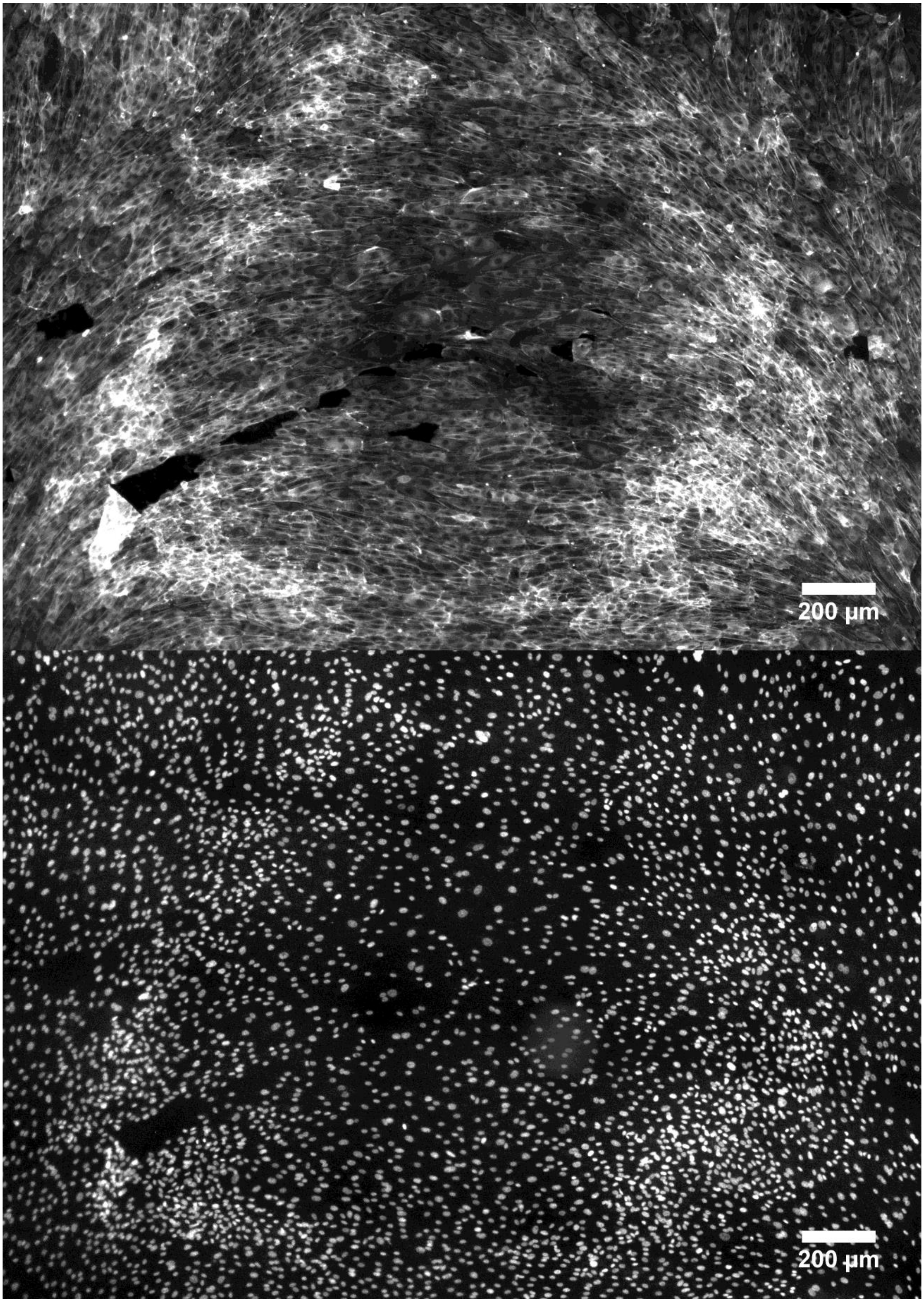
200 µm
200 µm

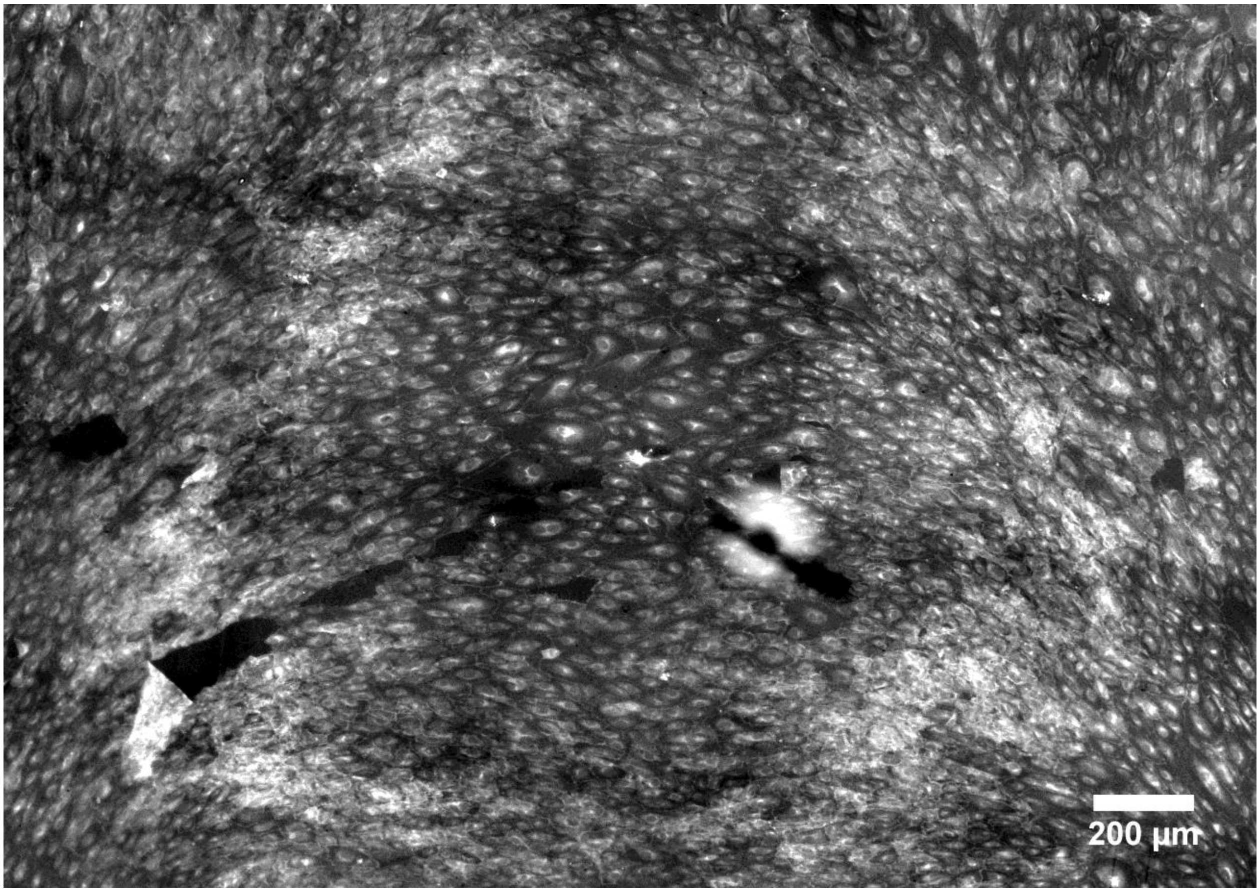
200 µm

Supplementary Video 1: Bioreactor Design. The components and the assembly of the bioreactor are illustrated, as well as the membrane in its flat and inflated state.

Supplementary Video 2: Dynamic CFD model of reactor membrane and corresponding WSS.

Supplementary Video 3: Live imaging of HUVECs on reactor membrane. 8% stretch, ~1 Hz, 0.4 l/min flow. The membrane is imaged 2 mm from the inlet edge, where the maximum WSS is created. As the physiological conditions are not exceeded, the full coverage is maintained. Note that live imaging upon cyclic deformation cannot provide optimal sharpness for the entire 3D region displayed. Therefore the cell layer may be slightly out of focus in some regions.

Supplementary Video 4: Live imaging of HUVECs on reactor membrane. 8% stretch, ~1 Hz, 0.6 l/min flow. The membrane is imaged 2 mm from the inlet edge, where the maximum WSS is created. As the physiological conditions are exceeded, HUVECs detach from the membrane in the area of maximum WSS.
